# Effect of moderate to high intensity aerobic exercise on blood pressure in young adults: The TEPHRA open, two-arm, parallel superiority randomized clinical trial

**DOI:** 10.1016/j.eclinm.2022.101445

**Published:** 2022-05-13

**Authors:** Wilby Williamson, Adam James Lewandowski, Odaro John Huckstep, Winok Lapidaire, Alexander Ooms, Cheryl Tan, Afifah Mohamed, Maryam Alsharqi, Mariane Bertagnolli, William Woodward, Cameron Dockerill, Annabelle McCourt, Yvonne Kenworthy, Holger Burchert, Aiden Doherty, Julia Newton, Henner Hanssen, John Kennedy Cruickshank, Richard McManus, Jane Holmes, Chen Ji, Sharon Love, Elena Frangou, Colin Everett, Melvyn Hillsdon, Helen Dawes, Charlie Foster, Paul Leeson

**Affiliations:** a Radcliffe Department of Medicine, Oxford Cardiovascular Clinical Research Facility Division of Cardiovascular Medicine, John Radcliffe Hospital, University of Oxford, Oxford OX3 9DU, UK; b School of Medicine, Trinity College Dublin, Dublin, Ireland; c Radcliffe Department of Medicine, Oxford Centre for Clinical Magnetic Resonance Research, Division of Cardiovascular Medicine, University of Oxford, Oxford, UK; d Department of Biology, United States Air Force Academy, CO, USA; e Nuffield Department of Orthopaedics, Centre for Statistics in Medicine, Rheumatology and Musculoskeletal Sciences, University of Oxford, UK; f Department of Diagnostic Imaging & Applied Health Sciences, Faculty of Health Sciences, Universiti Kebangsaan Malaysia, Kuala Lumpur, Malaysia; g Hôpital du Sacré-Cœur de Montréal Research Center (CIUSSS Nord-de-l’Île-de-Montréal), School of Physical and Occupational Therapy, McGill University, Montréal, Canada; h Nuffield Department of Population Health, BHF Centre of Research Excellence, University of Oxford, UK; i Nuffield Department of Orthopaedics, Rheumatology and Musculoskeletal Sciences, University of Oxford, UK; j Department of Sport, Exercise and Health, University of Basel, Switzerland; k School of Lifecourse Sciences, Kings College London, London, UK; l Nuffield Department of Primary Health Care Sciences, University of Oxford, Oxford, UK; m Warwick Clinical Trials Unit, University of Warwick, Warwick, UK; n MRC Clinical Trials Unit, University College London, London, UK; o Clinical Trials Research Unit, University of Leeds, Leeds, West Yorkshire, UK; p Sport and Health Sciences, University of Exeter, Exeter, UK; q Faculty of Health and Life Sciences, Oxford Brookes University, Oxford, UK; r School of Policy Studies, University of Bristol, Bristol, UK

**Keywords:** Hypertension, Prevention, Cardiovascular risk, Blood pressure, High blood pressure, Lifestyle intervention, Exercise, Aerobic training, Young adults

## Abstract

**Background:**

Exercise is advised for young adults with elevated blood pressure, but no trials have investigated efficacy at this age. We aimed to determine whether aerobic exercise, self-monitoring and motivational coaching lowers blood pressure in this group.

**Methods:**

The study was a single-centre, open, two-arm, parallel superiority randomized clinical trial with open community-based recruitment of physically-inactive 18–35 year old adults with awake 24 h blood pressure 115/75mmHg-159/99 mmHg and BMI<35 kg/m^2^. The study took place in the Cardiovascular Clinical Research Facility, John Radcliffe Hospital, Oxford, UK. Participants were randomized (1:1) with minimisation factors sex, age (<24, 24–29, 30–35 years) and gestational age at birth (<32, 32–37, >37 weeks) to the intervention group, who received 16-weeks aerobic exercise training (three aerobic training sessions per week of 60 min per session at 60–80% peak heart rate, physical activity self-monitoring with encouragement to do 10,000 steps per day and motivational coaching to maintain physical activity upon completion of the intervention. The control group were sign-posted to educational materials on hypertension and recommended lifestyle behaviours. Investigators performing statistical analyses were blinded to group allocation. The primary outcome was 24 h awake ambulatory blood pressure (systolic and diastolic) change from baseline to 16-weeks on an intention-to-treat basis. Clinicaltrials.gov registered on March 30, 2016 (NCT02723552).

**Findings:**

Enrolment occurred between 30/06/2016-26/10/2018. Amongst the 203 randomized young adults (*n* = 102 in the intervention group; *n* = 101 in the control group), 178 (88%; *n* = 76 intervention group, *n* = 84 control group) completed 16-week follow-up and 160 (79%; *n* = 68 intervention group, *n* = 69 control group) completed 52-weeks follow-up. There were no group differences in awake systolic (0·0 mmHg [95%CI, -2·9 to 2·8]; *P* = 0·98) or awake diastolic ambulatory blood pressure (0·6 mmHg [95%CI, -1·4. to 2·6]; *P* = 0·58). Aerobic training increased peak oxygen uptake (2·8 ml/kg/min [95%CI, 1·6 to 4·0]) and peak wattage (14·2watts [95%CI, 7·6 to 20·9]) at 16-weeks. There were no intervention effects at 52-weeks follow-up.

**Intepretation:**

These results do not support the exclusive use of moderate to high intensity aerobic exercise training for blood pressure control in young adults.

**Funding:**

Wellcome Trust, British Heart Foundation, National Institute for Health Research, Oxford Biomedical Research Centre.

**Research in context**

**Evidence before this study**

We completed a search from inception up to June 2019 with no language restrictions (PubMed, Scopus Web of Science, Cochrane, MEDLINE, EMBASE, CINAHL). Search terms included but were not limited to “physical activity”, “exercise”, “aerobic exercise”, “blood pressure” and “hypertension”. Our first published meta-analysis suggested scarce evidence on the effects of exercise on blood pressure in younger adults and the observed pooled intervention effect on blood pressure reduction was short-term (6 months) and dominated by studies with complex interventions including exercise, dietary and weight loss components. Our second published meta-review indicated variation in blood pressure response to type of exercise and level of blood pressure and that the young adult age group remained under-investigated.

**Added value of this study**

To the best of our knowledge, this is the first trial to exclusively investigate effectiveness of aerobic exercise intervention to lower blood pressure in young adults. The study design removes the confounding of dietary and weight loss intervention observed in previous studies and provides resting and ambulatory blood pressure measures up to 1 year following exercise intervention in young adults.

**Implications of all the available evidence**

Aerobic exercise may not be a reliable first line intervention to target blood pressure reduction in young adults. The available evidence suggests need for personalisation of intervention with short-term tracking and refinement of lifestyle intervention dependent on blood pressure response. Overall, further research is required to improve understanding of how best to lower blood pressure in young adults.

Alt-text: Unlabelled box

## Introduction

Based on current guidelines, up to 40% of young adults may have elevated blood pressure^1^^,^^2^ and seek medical advice to optimise their health.^3^ Young adult blood pressure predicts future risk of cardiovascular events^4^^,^^5^ and, if untreated, up to 50% of those with elevated blood pressure may transition to hypertensive levels in as little as 5 years.^2^^,^^5^^,^^6^ The benefits of antihypertensive medications in young adults with elevated blood pressure are unclear^7^ and therefore first-line management is typically lifestyle intervention with advice to increase aerobic exercise.^8^ However, surprisingly, no trials have investigated whether aerobic exercise training is effective at lowering elevated blood pressure levels in young adults^9^ and recent expert meta-reviews have identified aerobic exercise may not be as effective for blood pressure control in early stages of hypertension.^10^^,^^11^ Our systematic review of exercise interventions for blood pressure identified a bias in recruitment towards adults over 40 years of age with an intervention effect dominated by complex behavioural trials that included weight loss and nutritional strategies.^9^ Young adult hypertension may have a distinct pathophysiology and risk factor profile to older adult hypertension and aerobic exercise training has been shown to increase vascular and sympathetic tone in younger people, which, paradoxically, might increase blood pressure.^12^^,^^13^ Furthermore, up to a third of young adults with elevated blood pressure report a distinct *in utero* developmental history including preterm birth or pregnancy hypertension, which have been related to unique cardiovascular phenotypes.^14^, ^15^, ^16^, ^17^, ^18^, ^19^, ^20^, ^21^ The Trial of Exercise to Prevent HypeRtension in young Adults (TEPHRA) randomized clinical trial (RCT) was conducted to determine whether moderate to high intensity aerobic exercise, supported by physical activity self-monitoring and motivational coaching, significantly lowers blood pressure in physically inactive young adults with elevated blood pressures and a range of birth histories compared with a control group signposted to hypertension and lifestyle education materials.

## Methods

### Study design

The study was a single-centre, open, two-arm, parallel superiority randomized (1:1) controlled trial with open community-based recruitment. The study took place in the Cardiovascular Clinical Research Facility, John Radcliffe Hospital, Oxford, UK. The trial protocol^22^ and any subsequent amendments were approved by the University of Oxford as host institution and study Sponsor and the South Central Research Ethics Committee (REC) for the National Health Service Health Research Authority (NHS HRA) (Reference 16/SC/0016).^22^

### Participants

Persons were eligible for inclusion if they were: 18 to 35 years old; had 24 h awake ambulatory systolic and/or diastolic blood pressure >115/75 mmHg, but <159/99 mmHg; had a body mass index <35 kg/m^2^; were not on, and had not previously been prescribed, hypertension medications; had a verifiable birth history of preterm birth (<37 weeks) or full-term birth (≥37 weeks); and had the ability to access and use a computer and the internet. Participants could not take part: if they were pregnant; if they participated in structured exercise more than once per week or maintained high cardiovascular fitness; if they were unable to provide consent; had any contra-indications to exercise; were unable to walk briskly on the flat for 15 min; had any evidence of cardiomyopathy, inherited cardiac abnormalities or other significant cardiovascular disease. Participants were recruited through invitation from hospital birth registers, GP records, open recruitment, targeted online advertising via Facebook, Instagram and Twitter, and invitation following participation in previous studies. Participants gave written informed consent. Enrolment occurred between June 30, 2016, and October 26, 2018; final follow-up January 9, 2020.

### Randomization and masking

Following completion of baseline study measures, participants were randomly assigned through minimisation with a probabilistic element using an online randomization program (Sealed Envelope™) to one of two groups in a 1:1 ratio – a control group or an aerobic training and physical activity intervention. Minimisation factors were sex, age (<24, 24–29, 30–35 years) and gestational age at birth (<32, 32–37, >37 weeks). Due to the nature of the intervention, participants and those delivering the intervention were not masked. The outcome assessors were not involved in randomisation or intervention delivery and remained blinded until after completion of final analysis.

### Procedures

Participants were asked to complete three aerobic training sessions per week, aiming for 60 min exercise at 60–80% peak heart rate measured at baseline. Participants were encouraged to attend supervised sessions offered by the study team. The intervention replicates strategies identified during systematic review of exercise intervention that reduced blood pressure in older age groups.^9^ The intervention team included physiologists, physiotherapists, clinical nurse specialists and a physician, all trained in motivational coaching. Participants were gifted a wrist-worn heart rate and activity monitor (Fitbit Charge HR) and encouraged to wear daily with a goal of 10,000 steps per day.^23^ Activity monitors support self-monitoring, goal setting and regular feedback, which have been identified as effective physical activity behaviour change strategies.^24^^,^^25^ On completion of 16 weeks of training, participants received a 60 min motivational coaching session. Participants were asked to reflect on their experience of physical activity and the aerobic exercise intervention and encouraged to set long-term physical activity goals. The coaching session explored confidence and motivation to maintain regular physical activity and encouraged participants to identify personalised strategies to achieve their goals and maintain cardiovascular fitness until 52-week follow-up. To track physical activity in the intervention group, records were kept of sessions attended and activity from the wrist-worn activity monitor tracked using the Fitabase data management platform (Fitabase, San Diego, USA). Participants in the control group were sign-posted to educational materials produced by the British Heart Foundation explaining hypertension, hypertension prevention and recommended lifestyle behaviours to maintain heart health. The target intervention exposure was 3 aerobic sessions per week, completed on separate days, for 16 weeks, with a compliance threshold set at 80%; equivalent to ≥ 39 independent aerobic exposures with no greater than 2 weeks between exposures. Before recruitment commenced a compliant session was defined as a supervised exercise session, but during the course of the trial this definition was relaxed to include unsupervised sessions captured using the activity monitor. At baseline, at 16 weeks and 52 weeks post randomisation, participants completed lifestyle and physical activity questionnaires and underwent physical examinations, vascular measures, microvascular assessment, retinal vessel imaging, echocardiogram, blood sampling, cardiopulmonary exercise testing (CPET), 24 h ambulatory blood pressure, and seven day accelerometer. A subgroup underwent MRI at the baseline and 16 week visits.

### Outcomes

The primary outcome was change in 24 h ambulatory awake blood pressure (systolic and diastolic) from baseline to 16 weeks. Secondary outcomes included change in nocturnal and resting clinic blood pressure, change in peak cardiorespiratory fitness, peak exercise wattage, submaximal ventilatory anaerobic threshold, insulin resistance, cholesterol HDL ratio, glucose and vascular stiffness at 16 and 52 weeks. Additional secondary outcomes reported in the trial protocol will be presented in future manuscripts and include additional blood pressure, CPET and blood sample measures, as well as cardiac echocardiographic outcomes, cell culture data, wrist-worn accelerometery activity and walking speed, retinal imaging and in a subgroup of participants, dermal capillary measures, molecular and vascular cell function and heart, brain and liver MRI outcomes. Full details of the study objectives, outcomes and measurement procedures are detailed in the published study protocol.^22^ There were no changes to the study outcomes after publication of the study protocol. Participants were asked to report adverse events.

### Statistical analysis

To observe a treatment effect on systolic blood pressure of 5 mmHg powered to 80% (*p* = 0.05) required a total sample of 164 participants and to observe a similar effect on diastolic blood pressure required a sample of 114 participants, based on pooled SD from previous systematic review of 11 mmHg for systolic and 8 mmHg for diastolic pressures.^9^ Sample size was therefore set at 200 participants (100 in each group) to allow for 18% attrition. We did not adjust for multiple testing because the intervention was to be considered superior to the control (or vice versa) only if both systolic and diastolic blood pressure showed a significant difference. Participants were included in the analysis if they had data available for all relevant timepoints on an intention-to-treat basis. On completion of the study a total of 160 participants were included in the final analysis for the primary outcome. A priori significance levels of 0·05 and 95% confidence intervals are reported on two-sided hypothesis tests. All analysis was conducted in R Version 3.6.1.

Blood pressure measured at 16 and 52 weeks was analysed using analysis of covariance (ANCOVA) within a multivariate mixed effects model to estimate the primary outcome of change in blood pressure at 16 weeks. This model accounted for person as a random effect, and time (16 or 52 weeks), treatment, and the minimisation factors (sex, age, gestational age at birth) as covariates. Baseline blood pressure was included as a continuous variable. A treatment by time interaction was also included in the model to allow 16-week specific treatment effects to be calculated. This estimate of the 16-week treatment effect is the primary outcome estimate. The primary analysis was intention-to-treat (participants were analysed according to the group they were assigned irrespective of compliance) and the mixed model used all available data at each time point.

Secondary continuous outcomes analysed at 16 and 52 weeks were analysed using the same ANCOVA mixed effects model as the primary analysis but adjusting for their corresponding baseline variables.

Exploratory subgroup analyses according to gestational age, sex and BMI were performed using an interaction term to define the subgroups in an ANCOVA model with blood pressure measured at 16 weeks as the outcome (adjusted for baseline, minimisation factors, and treatment). These analyses were restricted to participants with non-missing subgroup data.

A sensitivity analysis was carried out using the per-protocol population including only those considered compliant as per the definition given in the intervention section. Post-hoc exploratory sub-group analysis was also performed to understand whether there was difference in effect related to volume of supervised or unsupervised exercise sessions completed. Two missing data sensitivity analyses on the primary analysis were performed. Details and results of these analyses are given in the supplementary material.

The trial statistician completed all primary, pre-specified 16-week and 52-week secondary, sensitivity and pre-specified sub-group analyses. Other 52-week secondary and post-hoc exploratory analyses were completed by a study team investigator blinded to participant identifier, with results verified by a second research team member. There was a data and safety monitoring committee. Clinicaltrials.gov registration number NCT02723552, prospectively registered March 30, 2016.

### Role of the funding sources

The funders had no role in the design and conduct of the study; collection, management, analysis, and interpretation of the data; preparation, review, or approval of the manuscript; and decision to submit the manuscript for publication. WW, AL, WL, and AO, JH had direct access to the data. All authors agreed on decision to submit for publication.

## Results

**Figure 1:**
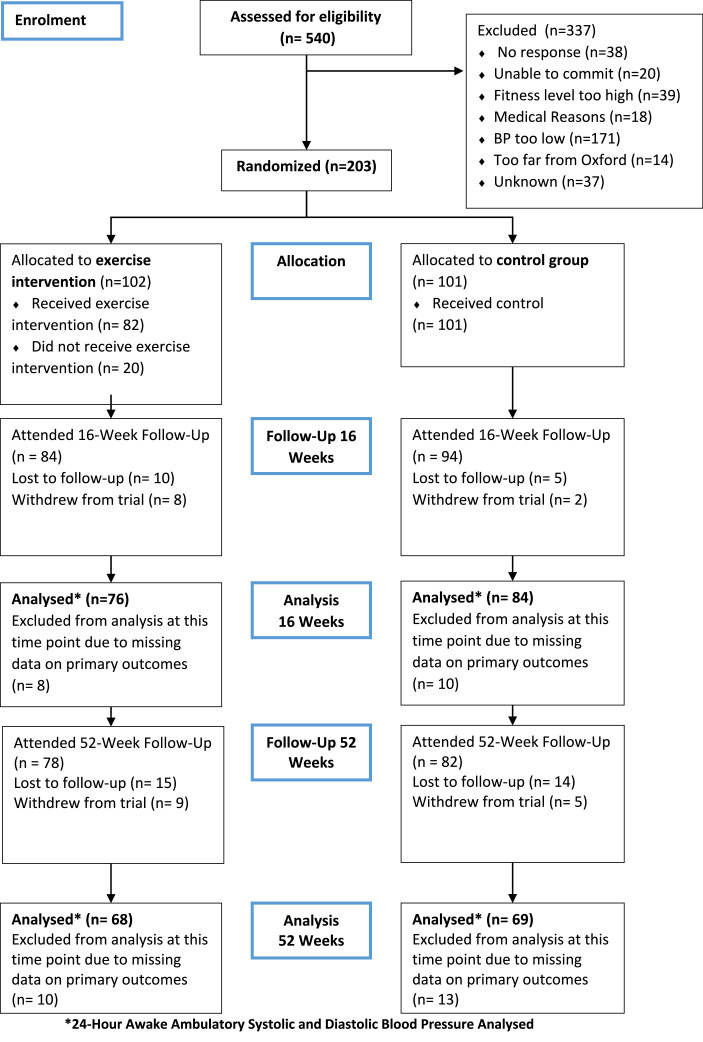
Trial profile.

Enrolment occurred between June 30, 2016, and October 26, 2018; final follow-up January 9, 2020. Baseline characteristics of participants are presented in Table 1. Of the 203 randomized participants, over 70% were white northern European (not shown) and 48% were male. At baseline, participants’ average vector magnitude, which is a proxy of overall activity, recorded during 7-day wear of a wrist-worn accelerometer averaged 29·15 mg (SD 6·52) in the intervention group and 29·48 mg (SD 7·83) in the control group^26^ (not shown). Mean baseline physical activity would be classified as sedentary with a projected age trajectory in highest cardiovascular risk group.^27^178 (88%) participants returned for 16-week follow-up of which the primary outcome was available on 160 (79%) participants. 160 participants returned for 52-week follow-up and 137 (67%) had awake ambulatory blood pressure data at 52 weeks. Reasons for the loss to follow-up at 16 weeks/52 weeks were: health reasons (1/1), lost contact (12/24), failed to contact (0/1) and moved away (2/3). There were 14 withdrawals in the trial (9 aerobic training, 5 control), 10 of these before the 16-week follow-up. Reasons for withdrawal included participant decision (5), pregnancy (5), and health reasons (2). Flow through the study can be seen in Figure 1.

**Table 1:** Baseline characteristics of the participants.

Characteristic	Exercise Intervention (*N* = 102)	Control (*N* = 101)
Age - years - Mean (SD)	27·7 (4·2)	27·8 (4·1)
Male – no. (%)	49 (48·0)	48 (47·5)
Preterm – no. (%)	26 (25·5)	26 (25·7)
Unemployed – no. (%)	1 (1·0)	2 (2·0)
University degree – no. (%)	76 (74·5)	79 (78·2)
Family history of high blood pressure, stroke, or heart attack – no. (%)	48 (47·1)	41 (40·6)
Body-mass index – kg m^−2^- Mean (SD)	25·2 (3·7)	24·9 (3·4)
Smoker – no. (%)	10 (9·8)	11 (10·9)
Alcohol - units per week - Mean (SD)	5·4 (5·6)	6·2 (8·5)
24 h awake ambulatory systolic blood pressure – mmHg - Mean (SD)	129·1 (9·3)	128·2 (8·7)
24 h awake ambulatory diastolic blood pressure – mmHg - Mean (SD)	77·0 (6·4)	77·4 (7·5)
Oxygen uptake peak – ml kg^−1^ min^−1^ Mean (SD)	33·0 (7·2)	34·7 (7·4)
Cholesterol-HDL ratio – mmol l^−1^ - Mean (SD)	3·3 (1·1)	3·3 (1·4)
Glucose – mmol l^−1^ - Mean (SD)	4·8 (0·6)	4·9 (0·5)
HOMA insulin resistance - Mean (SD)	1·1 (0·8)	1·0 (0·8)

Abbreviations: HDL = high density lipoprotein; HOMA = homeoostatic model assessment.

A total of 82 of 102 participants allocated to exercise received the intervention (79%). Activity data over the 52 weeks is not shown but across the 82 exercising participants the median volume of supervised moderate to vigorous exercise per week was 140 min per participant (IQR 110 to 160) at an intensity of 76% (SD 5) of maximum heart rate. Median daily wear of the Fitbit Charge HR was 105 days, with daily wear on 87% of available wear days across 16 weeks of the structured exercise programme. Using the Fitbit, participants registered a median of 337 min (IQR 213 to 462) of bouts of physical activity longer than 7 min at or above moderate intensity per week. The median daily step count in the first 16 weeks was 10,559 steps (IQR 9041 to 13,143). Total daily wear of the Fitbit dropped to less than 62% of available wear days between 16 and 52 weeks. In the last 60 days of the follow up period, total daily wear was 55% of available wear days and daily step count declined to a median of 9651 steps (IQR 7981 to 12,428). On average step count was 1442 steps per day (SD 2458) lower at the end of the 52 week follow up than during the 16 weeks structured exercise.

**Figure 2:**
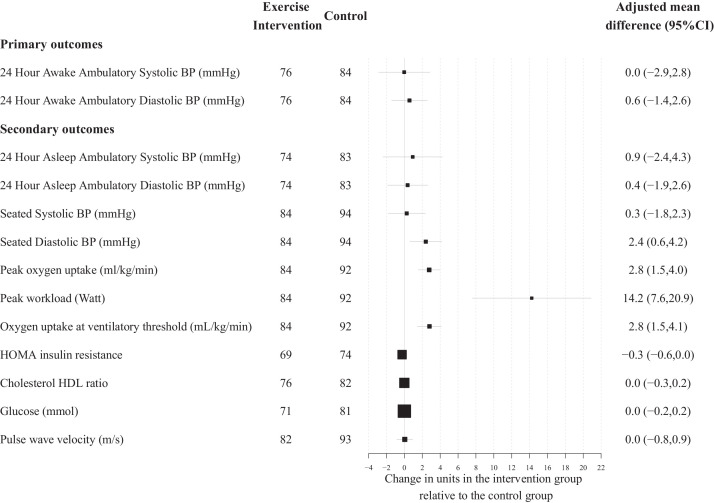
Differences between control and intervention for primary and secondary outcomes after 16 weeks moderate to high intensity exercise intervention. Change is presented as adjusted mean in the outcome of interest, in the units for that outcome, with 95% confidence interval error bars. A positive change represents an increase in the outcome measure in the intervention group. Abbreviations: BP = blood pressure, HOMA = homoeostatic model assessment, HDL = high density lipoprotein.

There was no evidence of a difference in awake systolic ambulatory blood pressure at 16-weeks (aerobic training *n* = 76 mean 126·9 mmHg [SD 11·5] vs control group *n* = 84 mean 125.8 mmHg [SD 10·0]; adjusted mean difference 0·0 [95% CI, −2.9 to 2·8]; *P* = 0·98). There were no between group differences in 16-week awake diastolic ambulatory blood pressure (aerobic training *n* = 76 mean 76·4 mmHg [SD 7·0] vs control group *n* = 84 mean 75·5 mmHg [SD 6·7]; adjusted mean difference 0·6 [95% CI, −1·4. to 2·6]; *P* = 0·58). (Table 2, Figure 2 and eFigure 5).

**Table 2:** Primary and secondary outcomes at baseline, 16 weeks and 52 weeks with adjusted mean difference between intervention and control groups at 16 and 52 weeks.

	Exercise intervention		Control		Adjusted Mean Difference (95% CI)	
	N	Mean (SD)	N	Mean (SD)		p-value
*Primary Outcomes*						
24 h Awake Ambulatory Systolic BP - mmHg						
Baseline	102	129·1 (9·3)	101	128·2 (8·7)	–	–
16 Weeks	76	126·9 (11·5)	84	125·8 (10·0)	0·0 (−2·9,2·8)	0·98
52 Weeks	68	127·8 (13·3)	69	125·5 (11·8)	1·5 (−1·6,4·6)	0·34
24 h Awake Ambulatory Diastolic BP - mmHg						
Baseline	102	77·0 (6·4)	101	77·4 (7·5)	–	–
16 Weeks	76	76·4 (6·9)	84	75·5 (6·7)	0·6 (−1·4,2·6)	0·58
52 Weeks	68	76·8 (8·3)	69	75·2 (7·8)	1·2 (−0·9,3·4)	0·26
*Secondary Outcomes*						
24 h Asleep Ambulatory Systolic BP - mmHg						
Baseline	99	108·1 (12·0)	99	107·9 (10·5)	–	
16 Weeks	74	109·2 (12·7)	83	108·4 (11·9)	0·9 (−2·4,4·3)	
52 Weeks	68	107·1 (10·2)	68	107·6 (12·7)	−0·9 (−4·5,2·7)	
24 h Asleep Ambulatory Diastolic BP -mmHg						
Baseline	99	61·3 (6·9)	99	61·2 (7·0)	–	
16 Weeks	74	62·5 (7·9)	83	62·2 (8·6)	0·4 (−1·9,2·6)	
52 Weeks	68	61·6 (6·0)	68	61·5 (8·2)	−0·3 (−2·7,2·1)	
Seated Systolic BP - mmHg						
Baseline	102	122·0 (11·5)	101	121·7 (10·2)	–	
16 Weeks	84	121·3 (10·7)	94	120·5 (10·2)	0·3 (−1·8,2·3)	
52 Weeks	79	121·1 (10·9)	82	120·6 (10·6)	0·7 (−1·5,2·9)	
Seated Diastolic BP - mmHg						
Baseline	102	75·7 (9·7)	101	76·9 (8·3)	–	
16 Weeks	84	76.6 (8.5)	94	74.3 (8.2)	2.4 (0.6,4.2)	
52 Weeks	79	75·8 (9·1)	82	75·9 (7·8)	0·3 (−1·6,2·2)	
Peak oxygen uptake - ml/kg/min						
Baseline	101	33·0 (7·2)	100	34·7 (7·4)	–	
16 Weeks	84	36·3 (7·0)	92	34·6 (6·7)	2·8 (1·5,4·0)	
52 Weeks	77	33·9 (7·5)	81	34·5 (7·3)	0·2 (−1·1,1·4)	
Peak workload - Watt						
Baseline	101	205·0 (50·5)	100	211·3 (55·9)	–	
16 Weeks	84	223·1 (49·6)	92	211·4 (53·2)	14·2 (7·6,20·9)	
52 Weeks	77	215·0 (50·1)	81	211·0 (53·1)	5·1 (−1·9,12·0)	
Oxygen uptake at ventilatory threshold - mL/kg/min						
Baseline	101	18·5 (5·8)	101	20·4 (6·7)	–	
16 Weeks	84	21·6 (6·1)	92	19·9 (5·9)	2·8 (1·5,4·1)	
52 Weeks	77	17·6 (5·6)	81	18·2 (5·6)	0·2 (−1·2,1·6)	
HOMA insulin resistance						
Baseline	90	1·1 (0·8)	84	1·0 (0·8)	–	
16 Weeks	69	0·9 (0·5)	74	1·2 (1·1)	−0·3 (−0·6,0·0)	
52 Weeks	63	1·1 (0·7)	67	1·0 (0·8)	0·0 (−0·3,0·3)	
Cholesterol HDL ratio						
Baseline	99	3·3 (1·1)	89	3·3 (1·4)	–	
16 Weeks	76	3·5 (1·0)	82	3·4 (1·0)	0·0 (−0·3,0·2)	
52 Weeks	72	3·6 (0·9)	72	3·4 (1·0)	0·2 (0·0,0·5)	
Glucose – mmol/l						
Baseline	94	4·8 (0·6)	91	4·9 (0·5)	–	
16 Weeks	71	4·8 (0·5)	81	4·8 (0·6)	0·0 (−0·2,0·2)	
52 Weeks	66	5·0 (0·8)	70	5·0 (0·5)	0·0 (−0·2,0·2)	
Pulse wave velocity - m/s						
Baseline	94	9·6 (1·3)	95	9·8 (1·6)	–	
16 Weeks	82	10·0 (2·8)	93	10·0 (3·1)	0·0 (−0·8,0·9)	
52 Weeks	78	10·6 (3·4)	80	10·5 (2·5)	0·2 (−0·8,1·1)	

Abbreviations: BP = blood pressure.

Cardiovascular fitness improved with aerobic training, demonstrated by an increase in peak oxygen uptake at 16 weeks (aerobic training *n* = 84 mean 36·3 ml/kg/min [SD 7·0] vs control group *n* = 92 mean 34·6 ml/kg/min [SD 6·7]; adjusted mean difference 2·8 [95% CI, 1·6 to 4·0]) and increase in peak wattage (aerobic training *n* = 84 mean 223·1 W [SD 49·6] vs control *n* = 92 mean 211·4 W [SD 53·2]; adjusted mean difference 14·2 [95% CI, 7·6 to 20·9]). At 52 weeks there were no between group differences for peak oxygen uptake or peak wattage. There was a larger submaximal oxygen uptake at the ventilatory anaerobic threshold (VAT) in the aerobic training group at 16 weeks (aerobic training *n* = 84 mean 21·6 ml/kg/min [SD 6·1] vs control group *n* = 92 mean 19·9 ml/kg/min [SD 5·9]; adjusted mean difference 2·8 [95% CI, 1·5 to 4·15]), this difference was not sustained at 52 weeks. There were no between group differences in 16-week or 52-week insulin resistance, cholesterol HDL ratio, blood glucose levels, or vascular stiffness (pulse wave velocity). There were also no differences for secondary measures of awake ambulatory blood pressure at 52 weeks or asleep ambulatory systolic and diastolic blood pressure, nor seated clinic systolic pressure, at 16 weeks or 52 weeks. Seated clinic diastolic blood pressure was higher in the intervention group at 16 weeks (aerobic training *n* = 84 mean 76·6 mmHg [SD 8·5] vs control *n* = 94 mean 74·3 mmHg [SD 8·2]; adjusted mean difference 2·4 mmHg [95% CI 0·6 to 4·2]) but this difference was no longer evident at 52 weeks (Table 2 and Figure 2).

**Figure 3:**
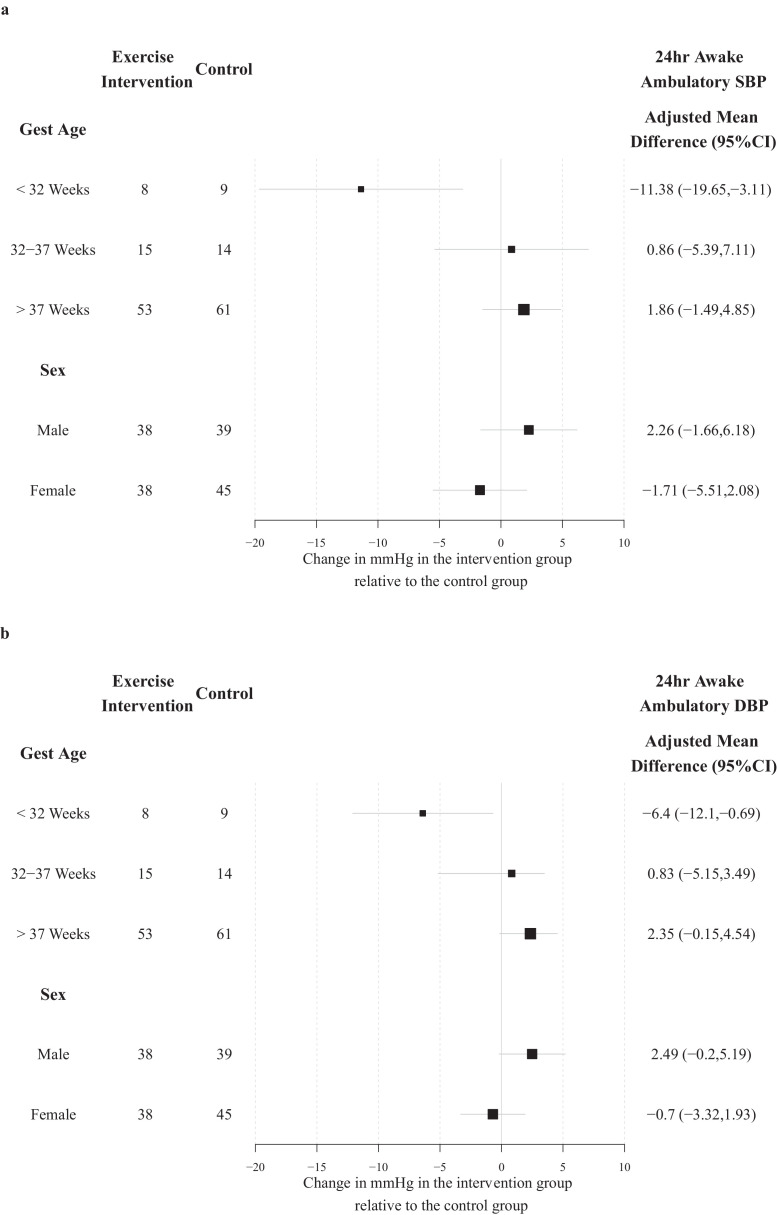
Pre-specified exploratory analysis of differences after16 weeks exercise intervention effect in awake systolic blood pressure (Panel A) and awake diastolic blood pressure (Panel B) according to subgroups based on gestational age at birth (<32 weeks, 32–37 weeks, >37 weeks) and gender. Change is reported as the adjusted mean difference in mmHg with error bars representing 95% confidence intervals. A positive change indicates an increase in the outcome in the intervention arm. Abbreviation: gest age = gestational age at birth.

In pre-specified, exploratory subgroup analysis, blood pressure response to intervention varied in relation to gestational age at birth (Figure 3). In the very preterm group, born before 32 weeks’ gestation (*n* = 17), both systolic and diastolic awake ambulatory blood pressure were lower at 16 weeks in the intervention group. Furthermore, awake ambulatory diastolic blood pressure was higher in those born full term after 37 weeks’ gestation. There were no significant differences between the randomised groups for other clinical subgroups (Supplement). Exploratory subgroup analysis found no difference related to whether the participant undertook supervised or unsupervised exercise sessions (Supplement).

There were two adverse events reported, both in the training group, an ankle sprain and toe injury. Injuries were self-limiting and activity adapted to allow continued participation in the intervention.

The per protocol analyses on awake systolic and diastolic blood pressure found no difference between groups at 16-weeks. The two sensitivity analyses completed to examine the robustness of the primary results against missing data showed no change from the main findings, implying the strategy used in the primary analysis was robust. Missing data results are presented in supplemental material.

## Discussion

In this trial involving young adults with elevated blood pressure, aerobic training and physical activity self-monitoring did not reduce ambulatory awake blood pressure compared to lifestyle education alone. The lack of effect was despite achieving an improvement in cardiovascular fitness in the training group. This is not in accordance with our hypothesis, which was based on previous studies that reported short-term benefit of aerobic exercise intervention on blood pressure.^9^ However, these training studies have tended to recruit older adults, included additional lifestyle interventions, and used clinic blood pressure readings. Our trial is the first to exclusively recruit younger adults with elevated blood pressure and to use ambulatory monitoring as the primary outcome. The intervention was also exclusively aerobic training. Following a recent meta-analysis, an expert consensus statement identified that the benefit of aerobic exercise appears to vary with stage of hypertension.^10^ Whether other modes of exercise such as dynamic or resistance training are more effective to lower blood pressure in younger adults now needs to be studied.^9^ Furthermore, the results demonstrate no additional benefit was obtained from currently available activity monitoring devices either during or after the intervention period. Alternative more complex, multicomponent lifestyle or pharmacological interventions require proper validation in younger adult populations.

The observed increase in cardiorespiratory fitness at 16 weeks is comparable to benefits observed in older adult hypertensive populations.^28^ The increase in fitness suggests the intervention was effective in terms of promoting cardiovascular adaptation. In comparison to age and sex matched reference data the relative mean increase was equivalent to offsetting a decade of decline in peak oxygen uptake or a shift in population mean from below lowest 20th percentile to a moderate level of fitness^29^^,^^30^ These gains would suggest a clinically meaningful increase in cardiorespiratory fitness, which was not evident in the control group who were just signposted to educational material. Therefore, independent of effects on blood pressure, the gain in fitness in the intervention group would be expected to reduce future cardiovascular risk. Secondary imaging outcomes involving the heart, brain and vasculature will be published subsequently to help inform whether exercise has resulted in remodelling likely to be of cardiovascular benefit in this young hypertensive population. However, if benefits are identified, the current results indicate they are not likely to be mediated through changes in blood pressure alone.^11^

The pre-specified exploratory subgroup analysis did indicate a potential heterogeneity in blood pressure response related to gestational age at birth. Those born very preterm had an improvement in both systolic and diastolic blood pressure profile at 16 weeks. By contrast, the intervention was associated with an increase in awake ambulatory diastolic blood pressure in those born at term, in keeping with the higher seated diastolic pressure after intervention in the whole group analysis. These findings would be consistent with cardiovascular differences described in young adults born peterm.^19^, ^20^, ^21^^,^^31^ Reassuringly, exercise intervention was observed to be safe in the study group, including for young adults born preterm. However, despite incorporation of evidence-based behavioural change strategies and physical activity wearables,^24^^,^^25^ benefits in fitness were not sustained at 52 weeks and the effects related to gestational age were no longer evident at this timepoint. Furthermore, due to the small sample sizes this exploratory analysis should be interpreted with caution unless replicated.

There were limitations to this single centre study. The study excluded participants with moderate to severe obesity so results may not be generalised to all young adult clinical populations. The wrist-worn device is commercially available and therefore the investigators did not have control over any additional features or behavioural prompting. The sample size calculation was adjusted for 18% loss to follow-up on 24 h ambulatory blood pressure. On completion of data cleaning ambulatory blood pressure was only available on 79% of participants at 16 weeks. Incomplete data related to lack of participant comfort during recording, disturbed sleep and reduced wear time. Whilst this means we did not reach our intended sample size, the study still had an overall power larger than 80% due to a smaller than expected observed standard deviation. Nevertheless, this study highlights compliance with ambulatory monitoring may limit feasibility and reliability as an outcome measure. Furthermore, the results could be biased in the event that data lost during follow-up were not missing at random but the sensitivity analyses do not suggest this is the case. Reassuringly, clinic blood pressure and cardiopulmonary exercise data was available on 88% of the randomized participants at 16 weeks and 79% of the randomized participants at 52 weeks. Due to the large number of secondary outcomes, there is a high chance of false positive results being discovered. Finally, it was also not possible to mask participants to trial group.

In conclusion, aerobic exercise training had no significant benefit in lowering awake ambulatory blood pressure in physically inactive young adults with elevated blood pressure compared to lifestyle education. Furthermore, benefits of aerobic training on other indicators of cardiovascular risk such as low submaximal and peak oxygen uptake only persisted during the aerobic exercise intervention. Motivational coaching and use of wrist-worn activity monitors had no sustained effects on maintenance of increased cardiovascular fitness achieved during training. Further work is required to understand whether different modes of exercise, alternative lifestyle interventions or pharmacological approaches are effective, safe and sustainable strategies to lower cardiovascular risk in young adults with elevated blood pressure.

## Contributors

WW wrote the first draft of the manuscript with input from all other authors in subsequent drafts. AO did the statistical analysis. All authors had full access to all the data in the study and had final responsibility for the decision to submit for publication. WW, AL, WL, and AO, JH have accessed and verified the data.

## Funding

Wellcome Trust, British Heart Foundation, National Institute for Health Research, Oxford Biomedical Research Centre.

## Data sharing statement

Trial data and materials are available to be shared subject to data sharing agreement and will be reviewed on a case by case basis. Please contact the corresponding author for requests.

## Declaration of interests

RM has received BP monitors for research from Omron and is working with them to develop a telemonitoring system. He does not personally receive payment for this. The other study investigators declare no competing interests.

## Appendix Supplementary materials

Image, application 1
