# Supplementary material for: Effect of moderate to high intensity aerobic exercise on blood pressure in young adults: The TEPHRA open, two-arm, parallel superiority randomized clinical trial
Source: eClinicalMedicine. 2022 May 13;48:101445. doi: 10.1016/j.eclinm.2022.101445 (PMC9112102; doi:10.1016/j.eclinm.2022.101445)
Supplement: Supplementary file 1 [file mmc1.docx]

## Supplementary appendix

1. Sensitivity Analysis – Missing Data
2. Per-Protocol Analysis
3. Subgroup Analysis
4. Figures
5. Tables
6. Protocol

p.1

p.2

p.3

p.3
p.8
p.11

1. Sensitivity Analysis - Missing Data

In this section, we present two sensitivity analyses to examine the robustness of our conclusions from the primary analysis. Our primary analysis was performed using a complete case analysis, which assumes the data is missing at random (MAR), i.e. the unobserved data is from the same conditional distribution as the observed data. This seems to be a plausible assumption for this trial as the numbers lost to follow-up are reasonably low, there are roughly equal numbers of missing data across both arms. Whilst there is an imbalance of withdrawals across the two arms (more in the intervention), the numbers are small and the actual reasons for withdrawal seem to be mostly unrelated to the intervention.

Our two sensitivity analyses are:

- Impute quantiles. For each missing data point, impute the median value of the arm that patient belongs to and analyse using ANCOVA adjusting for baseline, minimisation factors and treatment allocation. Repeat the analysis on a population that has the 60th quantile imputed for one arm’s missing values and the 40th quantile for the other, then again using the 70th and 30th quantiles. We repeat the process but flipping the arms. In total 5 sensitivity analyses will be performed and the results will be displayed graphically. Due to the level of missingness (>20%), imputing values beyond the 70th and 30th quantiles will start to become meaningless as this will distort the data to an unhelpful degree.

- Multiple Imputation by Chained Equations.

The first sensitivity is to test the MAR assumption, by imputing different values for each arm’s missing data we examine how the treatment effect is affected by breaking the MAR assumptions to varying degrees. Multiple imputation using the analysis model assumes MAR (just as complete case analysis does) so the second analysis does not test this assumption. The second analysis, using multiple imputation, is included at the request of the DSMC and TSC for completeness and to back-up the main analysis’s findings.

The intention-to-treat analysis strategy used is a comprehensive examination of the conclusions drawn from the main analysis. For clarity, eTable 1 shows the values being imputed for each quantile for both Systolic and Diastolic Blood Pressure for the first sensitivity analysis.

In eFigure 1 and 2, we can see how the estimates of the adjusted mean difference change depending on the values we impute. As we use lower values to impute for the intervention arm and higher values in the control arm, the effect size creeps towards favouring the intervention, as one would expect. The opposite is also true, lower values in the control and higher values in the intervention makes the mean estimate trend towards favouring the control arm. When we impute the 70th quantile for the intervention and 30th quantile for the control we yield a statistically significant result favouring the control arm to a 5% significance level for both Systolic and Diastolic Blood Pressure.

This sensitivity analysis tells us that we would need to see a large violation of the MAR assumption assumed in the main analysis to render the results invalid and, given the reasons for missingness in the TEPHRA trial, this departure being a reality is unlikely.

For our second sensitivity analysis on missing data, we performed Multiple Imputation by Chained Equations (MICE). This method is widely used in research due to its ability to account for statistical uncertainty caused by the imputations by pooling multiple imputations and its flexibility to handle variables of varying types (binary or continuous). In our imputations, we have imputed using variables from our analysis model and not included other variables that may predict missingness. In this analysis we used 40 multiple imputations, analysed each “complete” dataset separately then pooled the results using the pool command from the mice package in R which averages the estimates of the complete data model, computes the total variance over the repeated analyses by Rubin’s rules (Rubin, D.B. (1987). Multiple Imputation for Nonresponse in Surveys. New York: John Wiley and Sons). Using 40 imputations minimises the error caused by the imputation process. We present the results as we did with the primary analysis - in eTable 2 and eFigure 3. As we have complete data for the primary outcome at baseline, no imputation was necessary and has been included in the eTable 2.

The results from this sensitivity analysis using Multiple Imputation by Chained Equations are very similar to the results from the primary analysis. The main, null, result from the study is shown to be a robust result when looking at departures from the MAR assumption and different approaches when handling missing data.

#### 2. Per-Protocol Analysis

A sensitivity analysis on the primary outcome has been carried out on a per-protocol basis to examine robustness of conclusions to different assumptions about departures from randomised policies. The target intervention exposure was 3 aerobic sessions per week, completed on separate days, for 16 weeks, with a compliance threshold set at 80%; equivalent to ≥ 39 independent aerobic exposures with no greater than 2 weeks between exposures. Before recruitment commenced a compliant session was defined as a supervised exercise session, but during the course of the trial this definition was relaxed to include unsupervised sessions captured using the activity monitor. The same mixed model was used for the primary outcomes accounting for person random effects, baseline (as a continuous covariate) and 4-month blood pressure values (or peak oxygen uptake), gender, age (<24, 24–29, >30 years old), and gestational age (<32, 32-37, >37 weeks) as fixed effects with treatment by 16 week time point interaction also included was used to analyse 24 Hour Awake Ambulatory Blood Pressure (Systolic and Diastolic) on the per-protocol population. The results are presented in eTable 3, nonparametric 95% confidence intervals around the unadjusted mean difference and p-values are not presented. The results from the per-protocol analysis reinforce the interpretation of the main trial results: that there is no difference between the Control and Exercise Intervention groups in 24 Hour Awake Ambulatory Blood Pressure at 16 or 52 weeks post-randomisation.

3. Subgroup Analysis

Subgroup analysis as per Gestational Age (divided using the cut-offs defined in the minimisation procedure: < 32 Weeks, 32 - 37 weeks, > 37 Weeks) and Sex (Male and Female). The analysis has been performed through fitting a linear model adjusting for baseline and minimisation factors and fitting an interaction term between treatment allocation and the variable being subsetted (gestational age and sex in two separate analyses). Complete case analyses have been performed and the numbers in each subgroup from the complete case population are reported in the forest plot in eFigure 4. Subgroup analysis looking at subgroups on BMI, Glucose Level, Arterial Stiffness and Left Ventricular Mass (from MRI sub-study). The cut-offs have been determined through taking the ceiling of the median at baseline for each variable with the exception of Glucose; as this variable has a much smaller range the cut off is the median rounded to one decimal place. A complete cases analysis has been performed and the numbers in each subgroup is reported. Post-hoc exploratory sub-group analysis was also performed to understand whether there was difference in effect related to volume of supervised or unsupervised exercise sessions completed (above or below 3 hours per week) presented in eTable 4.

**4. FIGURES**

eFigure 1. Adjusted mean difference in systolic blood pressure according to level of blood pressure imputed for missing values in the intervention and control arms. From left to right imputed values increase in the intervention arm and reduce in the control arm. Imputed values are based on levels expected in each quantile of the study population. Change is presented as adjusted mean difference in systolic blood pressure after 16 weeks moderate to high intensity exercise in mmHg with 95% confidence interval error bars


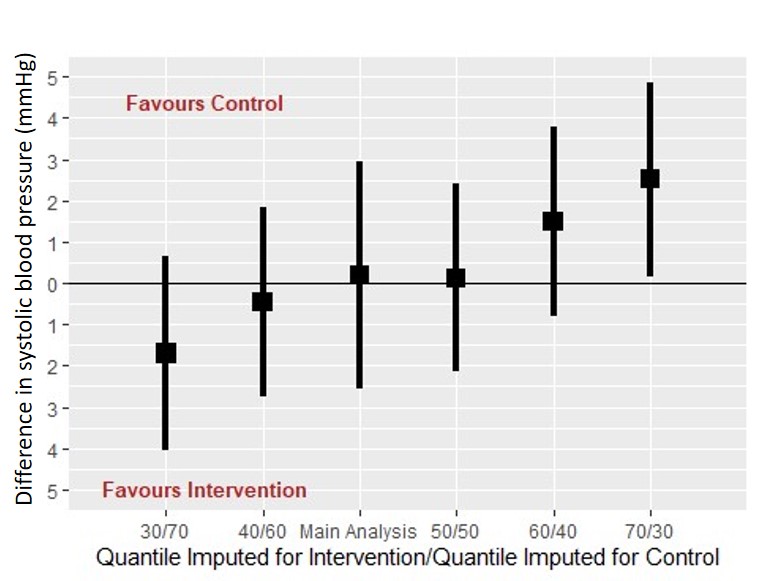


eFigure 2. Adjusted mean difference in diastolic blood pressure after 16 weeks moderate to high intensity exercise according to level of blood pressure imputed for missing values in the intervention and control arms. From left to right imputed values increase in the intervention arm and reduce in the control arm. Imputed values are based on levels expected in each quantile of the study population. Change is presented as adjusted mean difference in diastolic blood pressure after 16 weeks moderate to high intensity exercise in mmHg with 95% confidence interval error bars


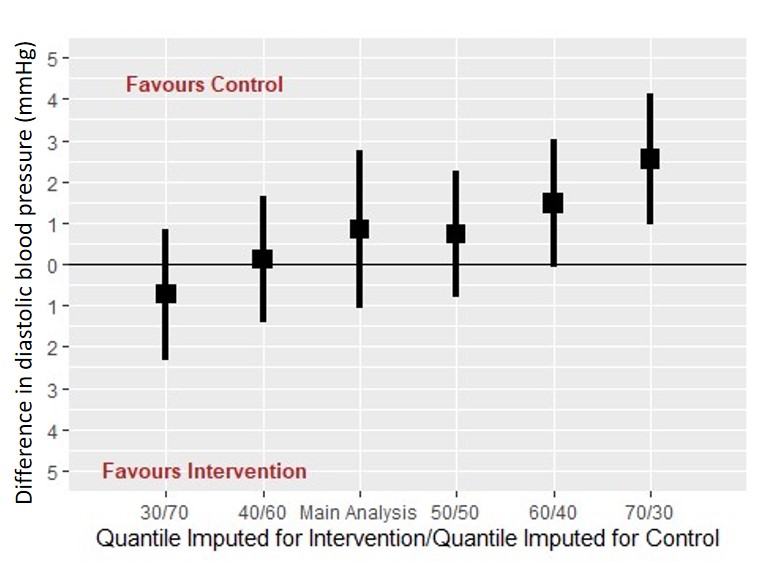


eFigure3. Sensitivity analysis using Multiple Imputation by Chained Equations (MICE) on the primary outcomes of 24hour awake ambulatory diastolic and systolic blood pressure. Change is presented as adjusted mean difference in each blood pressure variable between intervention and control groups after 16 weeks moderate to high intensity exercise in mmHg with 95% confidence interval error bars


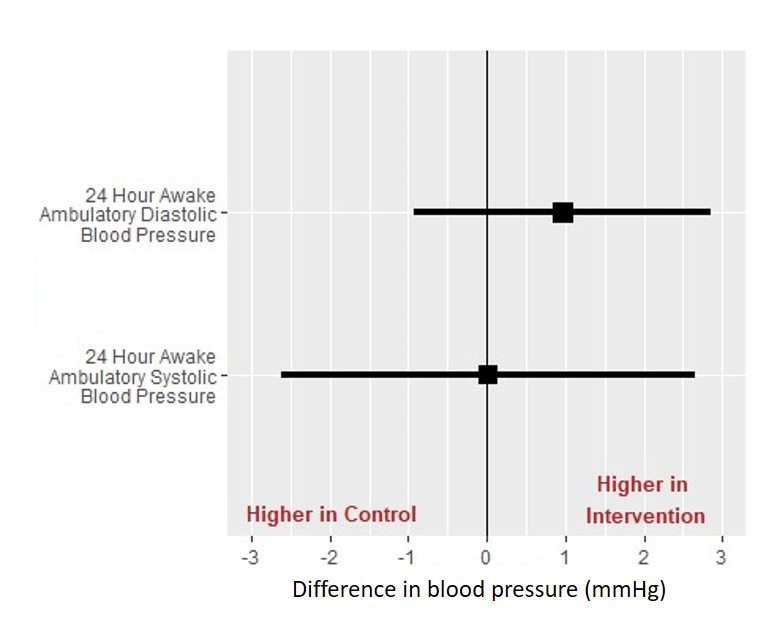


eFigure 4. Forest plots to demonstrate the adjusted mean difference in awake systolic ambulatory blood pressure (Panel A) and awake ambulatory diastolic blood pressure (Panel B) after a 16 week moderate to high intensity exercise intervention compared to a control group according to different subgroups of the study population. Subgroups are based on defined values of baseline body mass index, glucose level, arterial stiffness and left ventricular mass (for those who underwent magnetic resonance imaging) Change is reported as the adjusted mean difference in mmHg with errors bars representing 95% confidence intervals. Abbreviations: BMI = body mass index, LV = left ventricular, SBP = systolic blood pressure, DBP = diastolic blood pressure.

PANEL A. Awake Systolic Ambulatory Blood Pressure


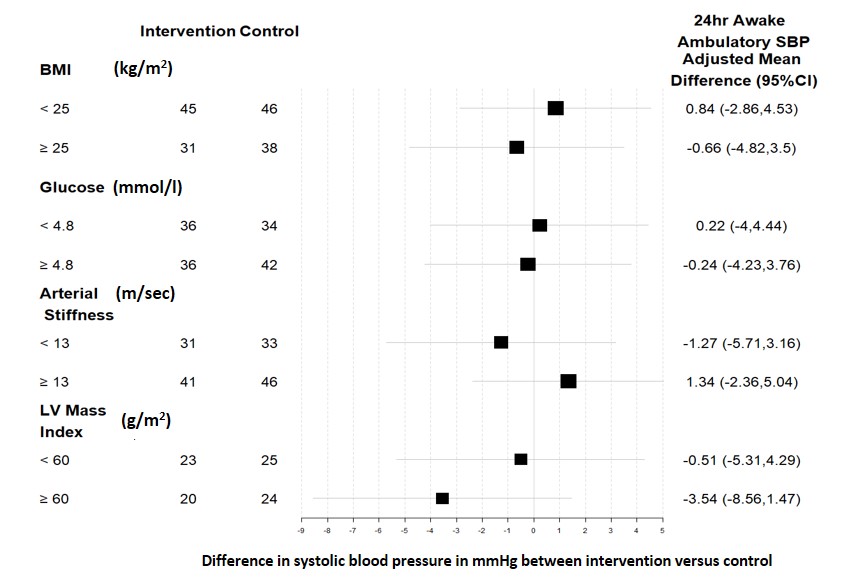


PANEL B. Awake Diastolic Ambulatory Blood Pressure

**
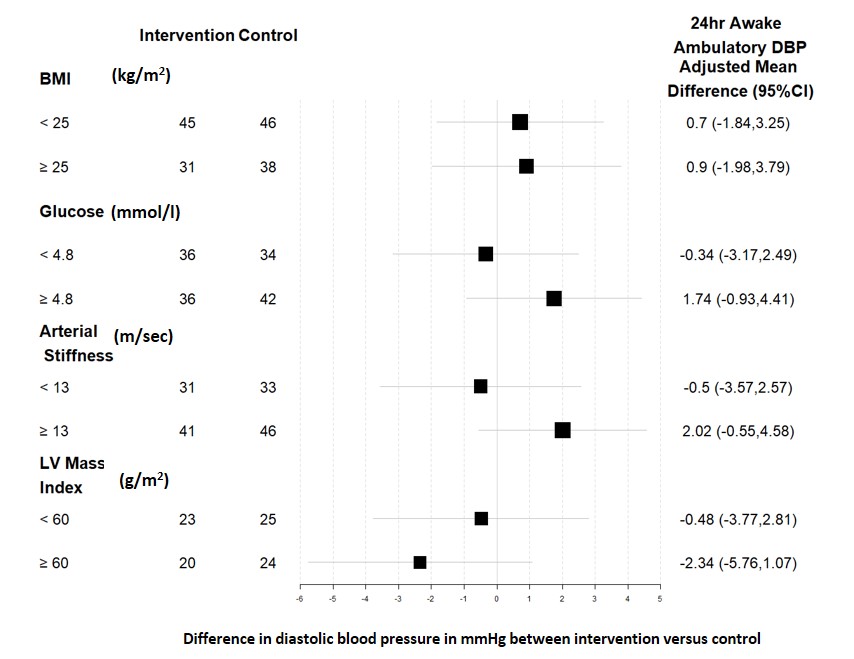
**

eFigure 5. Distributions of blood pressure within the study population before (left hand side) and after (right hand side) the 16 week moderate to high intensity exercise intervention. The distribution for the control and intervention arms are overlaid on each other within each graph. Abbreviation: BP=blood pressure


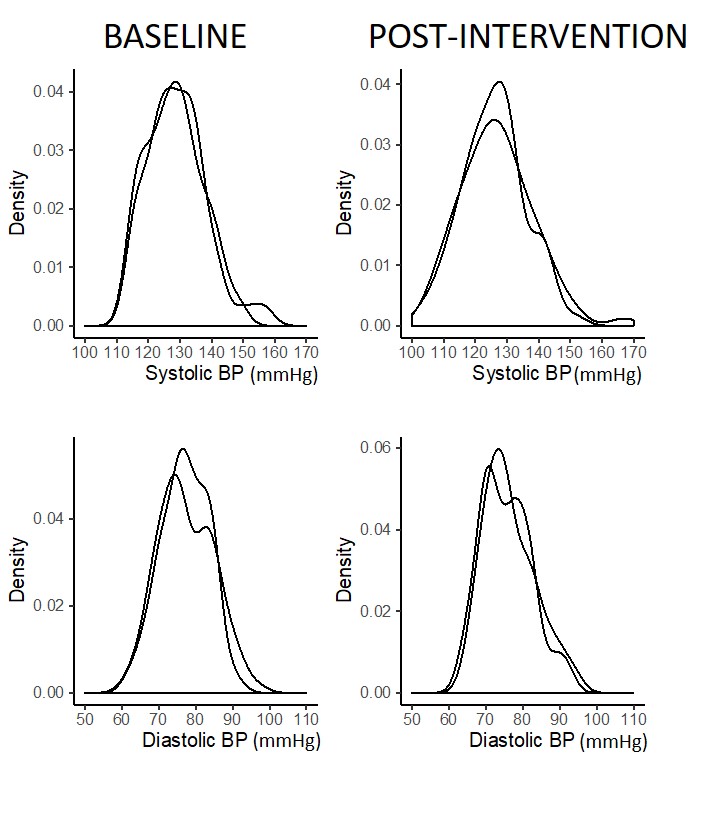


**5. TABLES**

| Quantiles | Systolic Blood Pressure |  | Diastolic Blood pressure |  |
| --- | --- | --- | --- | --- |
|  | Intervention | Control | Intervention | Control |
| 30 | 120 | 121 | 72 | 71 |
| 40 | 124 | 124 | 74 | 72 |
| 50 | 125 | 126 | 75 | 75 |
| 60 | 129 | 128 | 76 | 77 |
| 70 | 131 | 130 | 80 | 79 |

eTable 1. This table shows the values imputed for each quantile for both Systolic and Diastolic Blood Pressure for the first sensitivity analysis.

eTable 2. Sensitivity analysis using MICE

|  | Exercise Intervention | | Control | | Mean Difference (95%) | |
| --- | --- | --- | --- | --- | --- | --- |
|  | n | Mean (SD) | n | Mean (SD) | Unadjusted | Adjusted |
| **24 Hour Awake Ambulatory Systolic Blood Pressure** | | | | | |  |
| Baseline | 102 | 129.1 (9.3) | 101 | 128.3 (8.7) | - | - |
| 16 Weeks | 102 | 126.6 (11.3) | 101 | 126.0 (10.0) | 0.6 (0,1) | 0.0 (-2.6,2.7) |
| **24 Hour Awake Ambulatory Diastolic Blood Pressure** | | | | | |  |
| Baseline | 102 | 77.0 (6.4) | 101 | 77.4 (7.5) | - | - |
| 16 Weeks | 102 | 76.4 (6.9) | 101 | 75.6 (6.7) | - 1. (0,1) | 1. (-0.9,2.9) |

eTable 3. Per-protocol analysis

|  | Exercise Intervention | | Control | | Mean Difference (95%) | |
| --- | --- | --- | --- | --- | --- | --- |
|  | n | Mean (SD) | n | Mean (SD) | Unadjusted | Adjusted |
| **24 Hour Awake Ambulatory Systolic Blood Pressure** | | | | | | |
| Baseline | 82 | 129.41 (9.46) | 101 | 128.25 (8.68) | - | - |
| 16 Weeks | 71 | 126.93 (11.58) | 84 | 125.83 (10.00) | 1.10 | 0.02 (-2.87,2.92) |
| 52 Weeks | 63 | 127.81 (13.11) | 69 | 125.52 (11.82) | 2.30 | 1.52 (-1.61,4.65) |
| **24 Hour Awake Ambulatory Diastolic Blood Pressure** | | | | | | |
| Baseline | 82 | 77.48 (6.19) | 101 | 77.36 (7.46) | - | - |
| 16 Weeks | 71 | 76.51 (7.04) | 84 | 75.54 (6.73) | 0.97 | 0.61 (-1.41,2.64) |
| 52 Weeks | 63 | 77.01 (8.32) | 69 | 75.24 (7.76) | 1.77 | 1.31 (-0.88,3.49) |

eTable 4. Subgroup comparison of 24 Ambulatory Blood Pressure (mmHg) and Peak Oxygen Uptake (ml/kg/min) according to Volume of Weekly Supervised Exercise Completed.

|  | Exercise Intervention | | | Control | | Mean Difference (95%) | | | |
| --- | --- | --- | --- | --- | --- | --- | --- | --- | --- |
|  | n | Mean (SD) | | n | Mean (SD) |  | | | Adjusted |
| **24 Hour Awake Ambulatory Systolic Blood Pressure** | | | | | | | |  | |
| **Weekly Exercise Supervision ≥ 3 hours** | | | | | | | | | |
| Baseline | 45 | 129.6 (9.0) | | 101 | 128.3 (8.7) |  | | | |
| 16 Weeks | 43 | 127.7 (12.6) | | 84 | 125.8 (10.0) | -0.1 (-3.5,3.3) | | | |
| 52 Weeks | 35 | 129.2 (11.8) | | 69 | 125.5 (11.8) | 1.8 (-1.9,5.6) | | | |
| **Weekly Exercise Supervised < 3 hours** | | | | | | | | | |
| Baseline | 57 | | 128.8 (9.6) | 101 | 128.3 (8.7) | |  | | |
| 16 Weeks | 33 | | 125.9 (10) | 84 | 125.8 (10.0) | | 0.3 (-3.5, 4.1) | | |
| 52 Weeks | 33 | | 126.3 (14.8) | 69 | 125.5 (11.82) | | 1.3 (-2.7, 5.6) | | |
| **24 Hour Awake Ambulatory Diastolic Blood Pressure** | | | | | | | | | |
| **Weekly Exercise Supervision ≥ 3 hours** | | | | | | | | | |
| Baseline | 45 | | 77.4 (6) | 101 | 77.4 (7.5) | |  | | |
| 16 Weeks | 43 | | 77 (6.8) | 84 | 75.5 (6.7) | | 0.9 (-1.4,3.3) | | |
| 52 Weeks | 35 | | 78.3 (8.0) | 69 | 75.2 (7.8) | | 2.3 (-0.4,4.9) | | |
| **Weekly Exercise Supervision < 3 hours** | | | | | | | | | |
| Baseline | 57 | | 76.7 (6.7) | 101 | 77.4 (7.5) | |  | | |
| 16 Weeks | 33 | | 75.7 (7.2) | 84 | 75.5 (6.7) | | 0.3 (-2.4, 3.0) | | |
| 52 Weeks | 33 | | 75.2 (8.4) | 69 | 75.2 (7.8) | | 0.2 (-2.6, 4.9) | | |
|  |  | |  |  |  | |  | | |
| **Oxygen Uptake Peak** | | | | | | | | | |
| **Weekly Exercise Supervision ≥ 3 hours** | | | | | | | | | |
| Baseline | 45 | | 34.1 (7.8) | 100 | 34.7 (7.4) | |  | | |
| 16 Weeks | 45 | | 36.7 (7.6) | 92 | 34.6 (6.7) | | 3.0 (1.5, 4.5) | | |
| 52 Weeks | 41 | | 35.2 (8.3) | 81 | 34.5 (7.3) | | 0.7 (-0.9, 2.2) | | |
| **Weekly Exercise Supervision < 3 hours** | | | | | | | | | |
| Baseline | 56 | | 32.2 (6.6) | 100 | 34.7 (7.4) | |  | | |
| 16 Weeks | 39 | | 35.9 (6.2) | 92 | 34.6 (6.7) | | 2.4 (0.8, 3.9) | | |
| 52 Weeks | 36 | | 32.5 (6.2) | 81 | 34.5 (7.3) | | -0.6 (-2.2, 2.2) | | |
|  | | | | | | | |  | |

1. Protocol

**Study Title:**  Trial of Exercise to Prevent Hypertension in young Adults

**Internal Reference Number / Short title:** TEPHRA

**Ethics Ref:** 16/SC/0016 **IRAS Ref:** 194729

**Date and Version No:** 10th December 2020, Version 3.1

| **Chief Investigator:** | Professor Paul Leeson, PhD, FRCP  Professor of Cardiovascular Medicine and Consultant Cardiologist  Oxford Cardiovascular Clinical Research Facility  University of Oxford  Email: paul.leeson@cardiov.ox.ac.uk  Tel: 01865572846  Fax: 01865572840 |
| --- | --- |
| **Investigators:** | Dr Wilby Williamson, Division Cardiovascular Medicine, University of Oxford  Mr Odaro Huckstep, Division Cardiovascular Medicine, University of Oxford  Mrs Afifah Mohamed, Division Cardiovascular Medicine, University of Oxford  Ms Cheryl Mei Jun Tan, Division Cardiovascular Medicine, University of Oxford  Ms Charlotte Herdman, Division Cardiovascular Medicine, University of Oxford  Dr Adam Lewandowski, Division Cardiovascular Medicine, University of Oxford  Dr Jill Betts, Nuffield Dept of Clinical Neurosciences, University of Oxford  Dr Henry Boardman, Division Cardiovascular Medicine, University of Oxford  Prof Helen Dawes, Movement Science Group, Oxford Brookes University  Dr Aiden Doherty, Dept Population Health, University of Oxford  Prof Charlie Foster, Dept Population Health, University of Oxford  Ms Eleni Frangou, Centre for Statistics in Medicine, University of Oxford  Dr Stephen Gerry, Centre for Statistics in Medicine, University of Oxford  Prof Henner Hanssen, Institute of Sports Medicine, University of Basel  Mrs Yvonne Kenworthy Division Cardiovascular Medicine, University of Oxford  Dr David McCartney, Dept Primary Care, University of Oxford  Prof Richard McManus, Dept Primary Care, University of Oxford  Prof Stefan Neubauer, Division Cardiovascular Medicine, University of Oxford  Dr Julia Newton, NDORMS, University of Oxford |
| **Sponsor:** | University of Oxford |
| **Funder:** | Wellcome Trust and British Heart Foundation |
| **Chief Investigator Signature:** | The approved protocol will be signed by author(s) and/or person(s) authorised to sign the protocol |

No conflicts of Interest

**Confidentiality Statement**

This document contains confidential information that must not be disclosed to anyone other than the Sponsor, the Investigator Team, host organisation, and members of the Research Ethics Committee, unless authorised to do so.

**TABLE OF CONTENTS**

[1. KEY TRIAL CONTACTS 6](#_Toc10809254)

[2. SYNOPSIS 7](#_Toc10809255)

[3. ABBREVIATIONS 9](#_Toc10809256)

[4. BACKGROUND AND RATIONALE 10](#_Toc10809257)

[5. OBJECTIVES AND OUTCOME MEASURES 15](#_Toc10809258)

[6. TRIAL DESIGN 17](#_Toc10809259)

[7. PARTICIPANT IDENTIFICATION 20](#_Toc10809260)

[7.1. Trial Participants 20](#_Toc10809261)

[7.2. Inclusion Criteria (all participant apart from Validation sub-study) 21](#_Toc10809262)

[7.3. Exclusion Criteria (all participant apart from Validation sub-study) 22](#_Toc10809263)

[7.4. Inclusion criteria for TEPHRA blood validation sub-study 22](#_Toc10809265)

[7.5. Exclusion criteria for TEPHRA blood validation sub-study 23](#_Toc10809266)

[8. TRIAL PROCEDURES 24](#_Toc10809267)

[8.1. Recruitment 24](#_Toc10809268)

[8.2. Informed Consent 26](#_Toc10809269)

[8.3. Screening and Eligibility Assessment 26](#_Toc10809272)

[8.4. Randomisation 28](#_Toc10809273)

[8.5. Baseline Assessments 28](#_Toc10809274)

[Visit 1 (approx. 3 hours) 28](#_Toc10809275)

[Core Study Measures (n=200) 28](#_Toc10809276)

[Lifestyle and physical activity questionnaire (30 min) 28](#_Toc10809277)

[Physical Examination and ECG (15 mins) 29](#_Toc10809278)

[Cannulation and Blood Sampling (10 mins) 29](#_Toc10809279)

[24 hour Ambulatory Blood Pressure (ABP) 30](#_Toc10809280)

[7 day accelerometer 31](#_Toc10809281)

[8.6. Subsequent Visits 31](#_Toc10809283)

[Visit 2 (approx. 3 hours) 32](#_Toc10809284)

[Core Study Measures (n=200) 32](#_Toc10809285)

[Lifestyle and physical activity questionnaire (30 min) 32](#_Toc10809286)

[Physical Examination (15 mins) 32](#_Toc10809287)

[Cannulation and Blood Sampling (10 mins) 33](#_Toc10809288)

[24 hour Ambulatory Blood Pressure 33](#_Toc10809289)

[7 day accelerometer 34](#_Toc10809290)

[Visit 3 (approx. 1.5 hours) 34](#_Toc10809291)

[Core Study Measures (n=200) 34](#_Toc10809292)

[Lifestyle and physical activity questionnaire (30 min) 34](#_Toc10809293)

[Physical Examination (15 mins) 35](#_Toc10809294)

[Blood Sampling (10 mins) 35](#_Toc10809295)

[24 hour Ambulatory Blood Pressure 36](#_Toc10809296)

[7 day accelerometer 36](#_Toc10809297)

[Visit 4. Exit Interview 36](#_Toc10809298)

[8.7. Sub-Study Visits: Circulating biomarker validation and evaluation visit 1 and visit 2 37](#_Toc10809299)

[Recruitment of participants for TEPHRA blood validation sub-study 37](#_Toc10809300)

[Visit 1 37](#_Toc10809301)

[Consent (10 mins) 37](#_Toc10809302)

[Eligibility screening (5 mins) 37](#_Toc10809303)

[Study procedures 38](#_Toc10809304)

[Physical examination (10-15 mins) 38](#_Toc10809305)

[Blood sampling (10 mins) 38](#_Toc10809306)

[Visit 2 38](#_Toc10809307)

[8.8. Additional sample collection and handling 38](#_Toc10809308)

[8.9. Discontinuation/Withdrawal of Participants from Trial 38](#_Toc10809309)

[8.10. Definition of End of Study 39](#_Toc10809310)

[9. INTERVENTIONS 39](#_Toc10809311)

[Supervised Aerobic Exercise and Self-Monitoring Physical Activity Intervention 39](#_Toc10809312)

[(See Appendix E) 39](#_Toc10809313)

[10. SAFETY REPORTING 40](#_Toc10809314)

[10.1. Definition of Adverse Event (AE) 40](#_Toc10809315)

[10.2. Procedures for Recording Adverse Events 40](#_Toc10809316)

[10.3. Definition of a Serious Adverse Event 41](#_Toc10809317)

[10.4. Reporting Procedures for Serious Adverse Events 41](#_Toc10809318)

[10.5. Blood Draw and IV Placement 42](#_Toc10809319)

[10.6. MRI 42](#_Toc10809320)

[10.7. Exercise Training 44](#_Toc10809321)

[10.8. Cardiopulmonary Exercise Testing 45](#_Toc10809322)

[11. STATISTICS AND ANALYSIS 45](#_Toc10809323)

[11.1. Description of Statistical Methods 45](#_Toc10809324)

[11.2. The Number of Participants 45](#_Toc10809325)

[11.3. Analysis of Outcome Measures 47](#_Toc10809326)

[12. DATA MANAGEMENT 48](#_Toc10809327)

[12.1. Access to Data 48](#_Toc10809328)

[12.2. Data Recording and Record Keeping 48](#_Toc10809329)

[13. QUALITY ASSURANCE PROCEDURES 49](#_Toc10809330)

[14. ETHICAL AND REGULATORY CONSIDERATIONS 49](#_Toc10809331)

[14.1. MRI Imaging 49](#_Toc10809332)

[14.2. Storage of Blood Samples and Images 50](#_Toc10809333)

[14.3. Genetic Testing 50](#_Toc10809334)

[14.4. Cardiopulmonary Exercise Testing 50](#_Toc10809335)

[14.5. Exercise Associated Musculoskeletal Injury 50](#_Toc10809336)

[14.6. Use of Monitoring Devices 50](#_Toc10809337)

[14.7. Incidental Findings 51](#_Toc10809338)

[14.8. Declaration of Helsinki 51](#_Toc10809339)

[14.9. Guidelines for Good Clinical Practice 51](#_Toc10809340)

[14.10. Approvals 51](#_Toc10809341)

[14.11. Reporting 51](#_Toc10809342)

[14.12. Participant Confidentiality 51](#_Toc10809343)

[14.13. Expenses and Benefits 52](#_Toc10809344)

[14.14. Other Ethical Considerations 52](#_Toc10809345)

[15. FINANCE AND INSURANCE 52](#_Toc10809346)

[15.1. Funding 52](#_Toc10809347)

[15.2. Insurance 52](#_Toc10809348)

[16. PUBLICATION POLICY 52](#_Toc10809349)

[17. REFERENCES 53](#_Toc10809350)

[18. APPENDIX A: STUDY FLOW CHART 58](#_Toc10809351)

[19. APPENDIX B: STUDY STAGES and STUDY MEASURES 59](#_Toc10809352)

[20. APPENDIX C: SCHEDULE OF MEASURES and TIME ALLOCATION 60](#_Toc10809353)

[21. APPENDIX D: GANTT CHART 62](#_Toc10809354)

[22. APPENDIX E: PHYSICAL ACTIVITY INTERVENTION 62](#_Toc10809355)

[23. APPENDIX F: AMENDMENT HISTORY 63](#_Toc10809356)

# KEY TRIAL CONTACTS

| **Chief Investigator** | Professor Paul Leeson, PhD, FRCP  Professor of Cardiovascular Medicine and Consultant Cardiologist  Oxford Cardiovascular Clinical Research Facility  Level 1, John Radcliffe Hospital  Oxford, England. OX3 9DU  Email: paul.leeson@cardiov.ox.ac.uk  Tel: 01865572846 Fax: 01865572840 |
| --- | --- |
| **Sponsor** | University of Oxford  Clinical Trials and Research Governance  Joint Research Office 1^st^floor, Boundary Brook House Churchill Drive, Headington Oxford OX3 7GB  Email: ctrg@admin.ox.ac.uk |
| **Clinical Trials Unit** | Oxford Clinical Trials Research Unit  Phone 01865 223469  Email: octrutrialshub@ndorms.ox.ac.uk |
| **Statisticians** | Ms Eleni Frangou, Centre for Statistics in Medicine, University of Oxford  [eleni.frangou@csm.ox.ac.uk](mailto:eleni.frangou@csm.ox.ac.uk) +44 (0)1865 223453 |
| **Committees** | TRAIL STEERING COMMITTEE  Prof Helen Dawes, Movement Science Group, Oxford Brookes University  [hdawes@brookes.ac.uk](mailto:hdawes@brookes.ac.uk)  Prof Charlie Foster, Dept Population Health, University of Oxford  [charlie.foster@dph.ox.ac.uk](mailto:charlie.foster@dph.ox.ac.uk) +44 (0)1865 289241  Prof Richard McManus, Dept Primary Care, University of Oxford  [richard.mcmanus@phc.ox.ac.uk](mailto:richard.mcmanus@phc.ox.ac.uk) +44 (0)1865 617852  Dr Julia Newton, NDORMS, University of Oxford  [Julia.newton@ouh.nhs.uk](mailto:Julia.newton@ouh.nhs.uk)  Mr Colin Everett, Statistician, Leeds University  [C.C.Everett@leeds.ac.uk](mailto:C.C.Everett@leeds.ac.uk)  DATA MONITORING COMMITTEE:  Prof Melvyn Hillsdon, Sport & Health Sciences, University of Exeter  [M.Hillsdon@exeter.ac.uk](mailto:M.Hillsdon@exeter.ac.uk) +44 (0) 1392 722868  Prof Kennedy Cruickshank, Diabetes & Nutritional Sciences Div, King’s College  Tel: +44 (0) 20 7848 4270 Email: kennedy.cruickshank@kcl.ac.uk  Mr Chen Ji, Statistician, University of Warwick  [C.Ji.3@warwick.ac.uk](mailto:C.Ji.3@warwick.ac.uk)  TRIAL MANAGEMENT GROUP  Dr Wilby Williamson, Division Cardiovascular Medicine, University of Oxford  Mr Odaro Huckstep, Division Cardiovascular Medicine, University of Oxford  Ms Charlotte Herdman, Division Cardiovascular Medicine, University of Oxford  Ms Jenny Shaw, Oxfordshire Sport and Physical Activity Manager  Ms Fiona McMillan, Oxford Centre for Clinical Magnetic Resonance Research |

# SYNOPSIS

| **Study Title** | Trial of Exercise to Prevent Hypertension in young Adults | |
| --- | --- | --- |
| **Internal ref. no. / short title** | TEPHRA | |
| **Study Design** | Randomised Control Trial | |
| **Study Participants** | Young adults aged 18 to 35 years old with preterm birth (less than 37 weeks gestation) and full term birth (37 or more weeks gestation) histories, clinic systolic blood pressure of between 110 and 159 mmHg or diastolic pressure blood pressure between 70 and 99 mmHg and/or 24 hour awake ambulatory systolic blood pressure between 115 and 151 mmHg or diastolic pressure blood pressure between 75 and 96 mmHg. | |
| **Planned Sample Size** | 220 participants (100 preterm and 100 full term; 20 healthy participants) | |
| **Planned Study Period** | January 2016 to 31^st^ May 2021 | |
|  | **Objectives** | **Outcome Measures** |
| **Primary** |  |  |
|  | To compare the effect of physical activity and aerobic exercise intervention versus usual care/minimal intervention on awake blood pressure levels in young adults of varying gestational age with elevated blood pressure | Systolic and Diastolic blood pressure measured during 24 hour ambulatory blood pressure at baseline and upon completion of 16 week intervention |
| **Secondary** |  |  |
|  | To compare the sustained effect of physical activity and aerobic exercise intervention versus usual care/minimal intervention on awake blood pressure levels in young adults of varying gestational age with elevated blood pressure | Systolic and Diastolic blood pressure measured during 24hour ambulatory blood pressure at baseline, upon completion of 16 week intervention, and at and 52 weeks post-baseline |
|  | To compare the effect of physical activity and aerobic exercise intervention versus usual care/minimal intervention on cardiopulmonary exercise performance in young adults of varying gestational age with elevated blood pressure | Oxygen uptake and carbon dioxide exchange kinetics across submaximal and peak exercise |
|  | To compare the effect of physical activity and aerobic exercise intervention versus usual care/minimal intervention on central blood pressures and vascular stiffness in young adults of varying gestational age with elevated blood pressure | - Pulse wave velocity - Augmentation index - Central blood pressure |
|  | To investigate cardiac adaptation and remodelling following exercise intervention in preterm and full term born participants compared to non-exercising controls | Cardiac Echocardiography (all participants n=200)  -Cardiac Mass  -Ventricular Structure and Function  -3D-shape and functional analysis  Cardiac MRI (N=100)  -Cardiac Mass  -Ventricular Structure and Function  -3D-shape and functional analysis |
|  | To investigate the change in microvascular structure and function post exercise intervention compared to control | Retinal Imaging  Dermal Capillaroscopy |
|  | To access the degree of natural variability of circulating angiogenic and vascular biomarkers in the blood of healthy volunteers | Blood analysis tests including but not limited to protein and gene-expression assays |
|  | To quantify mean changes in circulating angiogenic and vascular markers and biomarkers at rest and following peak exercise between intervention and control participants | Whole, plasma, and serum blood samples at rest and following peak exercise testing |
|  | To investigate levels of vasoactive markers and biomarkers related to renal function at rest and following peak exercise between intervention and control participants | Whole, plasma, and serum blood samples at rest and following peak exercise testing |
|  | To investigate neurophysiological remodelling following exercise intervention compared to control | Brain MRI (N=100)  -Ventricular volume and shape  -Presence of white matter lesions  -White matter microstructural differences including fractional anisotropy  -Vessel Density and Tortuosity  Gait analysis accelerometry profile |
|  | To investigate hepatic remodelling and mean change in hepatic adiposity pre and post exercise intervention compared to control | Liver MRI (N=100)  Liver MRI Endpoints  Structure and volume  Intra-hepatic lipid content  Steatohepatitis  Hepatic fibrosis |
|  | To investigate the correlation between physical activity behaviour change and participants’ cognitive and psycho-social determinants of exercise including self-efficacy to exercise and motivations to exercise | Objective measure of physical activity (7 day wear of activity monitor)  Self-reported questionnaire responses, including self-reported physical activity questionnaires, cognitive and psycho-social questionnaire items and self-efficacy measures. |
| **Tertiary** |  |  |
|  | Participant experience: assessment of individual experience following intervention | Structured interview (participants) |
|  | Economic evaluation: assessment of feasibility for scaling intervention to multi-centre trial including intervention process evaluation | EQ-5D-5L health questionnaire results  Structured interviews (study team and personnel)  Review of medical notes/GP consultations |

# ABBREVIATIONS

| AE | Adverse Event |
| --- | --- |
| CI | Chief Investigator |
| CRF | Case Report Form |
| CTRG | Clinical Trials & Research Governance, University of Oxford |
| GCP | Good Clinical Practice |
| GP | General Practitioner |
| ICF | Informed Consent Form |
| NHS | National Health Service |
| PI | Principal Investigator |
| PIL | Participant/Patient Information Leaflet |
| R&D | NHS Trust R&D Department |
| RCT | Randomised Control Trial |
| REC | Research Ethics Committee |
| SOP | Standard Operating Procedure |

# BACKGROUND AND RATIONALE

**Early Hypertension**

Hypertension contributes to 9 million annual global deaths^1^. In the United Kingdom, 1 in 4 adults over the age of 25 are hypertensive and the incidence in the 18 to 40 year old population may be increasing^2^. North American population studies report incidence as high as 1 in 5 of the under-40 age group^3^. Targeted early treatment and primary prevention may have significant benefits on future morbidity and mortality. Blood pressure reductions of as little as 2 mmHg may be enough to reduce future stroke risk by 10%^4^. To drive improvement in primary prevention strategies more understanding is required of the effectiveness of lifestyle and behavioural intervention to lower blood pressure in adults with early cardiovascular risk. This includes investigation of the response to intervention in young adults known to have unique cardiovascular phenotypes associated with early life exposures and familial risk. Prevalence of hypertension is higher in young adults with history of preterm birth and exposure to maternal pregnancy complications such as pre-eclampsia^5–8^. Our recent work supports distinct aetiological pathways for this early cardiovascular risk^9^. The differences observed in blood pressure between preterm and full term born groups track from childhood and by early adulthood approach 10mmHg difference in systolic blood pressure, resulting in a 2-3 fold increase risk of early hypertension.^6,10,11^ In the UK 6-12% of the population are born preterm equivalent to 1-2 million young people at risk of early hypertension. This predicted cardiovascular risk burden is supported by our recent analysis of the Raine Western Australia longitudinal birth cohort, which identified that 1 in 3 adults diagnosed with hypertension before the age of 30 had been exposed to pregnancy complications^12^.

**Trial Rationale**

Physical activity promotion may be an attractive and acceptable strategy to improve cardiovascular health in young adults at risk of early hypertension and cardiovascular disease^13^. Our recent systematic review of exercise interventions demonstrated reductions of up to 5mmHg in systolic and diastolic blood pressure following supervised exercise. However, the review identified significant deficiencies in the investigation of exercise response in adults under 40 years of age. In addition, there is limited explanation of heterogeneity in blood pressure response following exercise training in the general population or across subgroups. Due to the unique cardiovascular phenotype of those born premature, it is uncertain whether, as adults, they will have the same blood pressure response and beneficial cardiovascular adaptation as observed in the wider population. The proposed randomised control trial will investigate the effects of exercise intervention on a young adult population with pre-hypertension or stage 1 hypertension. The trial includes a subgroup of participants born preterm and compares them to a control group born full term.

**Predicted cardiovascular exercise adaptations and support for proposed outcome measures**

- **Blood Pressure**

Our recent systematic review of RCTs delivering exercise interventions in young adults with prehypertension or stage 1 hypertension reported mean decrease of 5.4 mmHg and 5.43 mmHg in systolic and diastolic blood pressures following 3 to 6 months of supervised intervention. The proposed trial will be powered to achieve similar post interventions effects in blood pressure, reporting 24 hour ambulatory blood pressure as the primary outcome at 16 and secondarily at 52 weeks. 24 hour ambulatory blood pressure monitoring is the gold standard measure used in diagnosis and management of high blood pressure.

Our pilot data supports cardiovascular fitness as a mediator of lower blood pressure in adults with history of preterm birth or exposure to pregnancy complication. Systolic and diastolic blood pressures were 5mmHg and 10mmHg lower in preterm born adults with higher cardiovascular fitness, which we reported as exercise capacity above 30 ml/kg/min. The distinctions in blood pressure associated with fitness were more marked in preterm compared to full term born groups when applying the same criteria. This supports the hypothesis that adults with preterm birth and pregnancy complication may have lower resilience to the risks of sedentary behaviour and lower cardiovascular fitness.

- **Cardiac Structure and Function**

We have previously used 3-dimensional mesh analysis of cardiovascular magnetic resonance images to demonstrate prematurity is associated with significantly increased cardiac mass, shortened ventricles and reduced cavity size compared to adults born at term^14,15^. These features are associated with impaired systolic and diastolic myocardial deformation and 20% of the preterm cohort had right ventricular (RV) ejection fractions below the lower limit of the control group, with 6% fulfilling clinical criteria for RV dysfunction^14,15^. Cardiovascular adaptation following exercise training may lead to significant improvement across these parameters^13,16,17^. To meet increasing metabolic demand, cardiac output increases in response to exercise. This is associated with remodelling of the atria, ventricular walls and respective cavity sizes leading to increased end diastolic volumes and improved myocardial function. It is currently unknown whether these anticipated changes will be demonstrated in the preterm cardiovascular phenotype. To track potential changes in cardiac structure and function, study participants will undergo detailed cardiac ultrasound imaging and a subgroup of participants will complete detailed MRI imaging.

- **Microvascular Function and Angiogenesis**

We have observed a significantly altered circulating anti-angiogenic profile in adults born preterm, including elevations in soluble endoglin (5.64± 1.03 vs 4.06±0.85 ng/mL) and soluble fms-like tyrosine kinsase-1 (88.1±19.0 vs 73.0±15.3 pg/mL)^14^. Furthermore, these elevations are associated with reductions in capillary density (r=-0.57, p=0.002), which, in mediation analysis, accounted for increases in 24-hour systolic blood pressure (r=-0.55, p=0.01). Changes in peripheral capillary density and peripheral resistance are known to be an early adaptation to exercise^18,19^. Exercise-induced angiogenesis may be a key factor influencing cardiovascular adaptation and altering the risk profile of preterm born adults^20^. Angiogenic adaptation in response to exercise will be compared across the subgroups defined by gestational age. Angiogenic and vascular function markers and biomarkers will be measured from blood samples at baseline and follow-up, pre and post exercise testing and will be correlated with resting microvascular measures such as dermal capillaroscopy and retinal imaging.

- **Vasoactive Renal Function Analysis**

Renal function exerts a potent influence on blood pressure and vascular function through multiple mechanisms including moderating the Renin Angiotensin Aldosterone System (RAAS). Pharmaceutical blockade of angiotensin II and its pressor effect is an established and effective practice in clinical treatment of hypertension^21^. A recent systematic review linked exercise to reductions in angiotensin II levels^22^, though other research has suggested that angiotensin II levels may not be significantly altered by exercise^23^. Other research suggests that pre-eclampsia is associated with increased angiotensin II sensitivity^24^. However, angiotensin (1-7) appears to oppose some effects of angiotensin II and is associated with reduced blood pressure when orally administered to Spontaneously Hypertensive Rats^25^. In humans, reduced levels of angiotensin (1-7) have been linked to preterm birth^26^. Preliminary research also indicates that biomarkers may be able to play a role in characterizing RAAS function and activation^27^.

Characterizing markers and biomarkers related to RAAS activity may help to better understand the aetiology of hypertension in the trial groups and how this pathway is affected by chronic aerobic exercise.

**Neurophysiological adaptation**

Elevated blood pressure is a major risk factor for future cerebrovascular incidents^4^ and may be a more significant risk factor for preterm born adults and those exposed to pre-eclamptic pregnancy. Strikingly, the risk of stroke before the age of 50 years in adults exposed to pregnancy complications is increased 2-3 fold.^28^ Potential explanation for increased stroke and hypertensive risk is the unique pathophysiological vascular development during the perinatal period. Cerebral magnetic resonance (MR) angiography in neonates born preterm has demonstrated a significant developmental immaturity including reduced tortuosity in cerebral arteries, as well as sparser and less complex peripheral vessels^29^. Vascular density in the white matter has been shown to remain low compared with other brain regions^30^ and these differences may, in part, underlie reports of poor cerebral perfusion in the pre-term neonatal brain^31^. The observed differences in perfusion have been associated with regional hypoxia-ischaemia, particularly in the white matter, which is likely to ultimately predispose to white matter injury. The cerebral and retinal microvasculature share similar characteristics^32^, and clinically visible retinopathy signs are associated with an increased risk of stroke^33^. Subjects with early structural changes in retinal microvasculature are more likely to have cerebral microbleeds^34^, suggesting changes in retinal vascular health may reflect cerebrovascular health.

Physical activity is associated with a decreased stroke incidence^35,36^ and animal studies investigating brain damage after induced stroke show marked decreases in neuronal damage, infarct volume and improved survival in animals undergoing exercise preconditioning^37,38^. Exercise-induced angiogenesis may be a key factor in this response. An MR angiography study in older adults revealed a greater number of small vessels in the brains of aerobically active individuals compared with those less active^39^, and animal studies have repeatedly reported cerebral angiogenesis in response to physical exercise ^40,41^. Rats undergoing exercise preconditioning show a 10-fold increase in microvessel density in the striatum compared with controls, coupled with significantly reduced infarct volume and neurological deficits following induced stroke^38^. To track potential cerebral vascular adaptations to exercise, all participants will have retinal microvascular measures and a subgroup will have detailed cerebrovascular brain imaging. Our collaborators have been able to demonstrate optimal adaptation in the retinal microvascular in obese pre-hypertensive adults following exercise training^42^. The use of detailed MR angiography will allow for tracking of similar changes across the cerebral vascular beds.

**Exercise and Metabolic Health**

Risk of metabolic dysfunction is well established in preterm cohorts^11^ and highly correlated with cardiovascular risk in the general population. Hepatic structure and function may be a primary determinant of metabolic status^43^. A primary driver of optimal hepatic structure and function is believed to be cardiovascular fitness^44–46^. Improved cardiovascular fitness and reductions in fatty liver content are closely associated^46^. Further investigation is required to clarify if cardiovascular risk reduction and positive exercise training adaptation in young adult populations with pre-hypertension are correlated with hepatic structure and function. Detailed liver MRI will be completed on the subgroup of participants undergoing MRI imaging at baseline and follow-up.

**The purpose of the TEPHRA blood cell and biomarker validation sub-study**

Hypertension affects 1 in 4 adults in the UK and increases the risk of cardiovascular diseases and stroke. It is caused by a multitude of factors. As part of an ongoing trial, TEPHRA study is a randomised controlled trial to determine whether modification of lifestyle through an exercise intervention can reduce heart and blood vessel risk in young adults. In the sub-study, healthy participants will undergo measurements of specific characteristics of blood cells and circulating factors in venous blood obtained from veins as part of protocol validation. It is crucial to validate how blood cells and circulating factors vary naturally from day to day in the same individual.

# OBJECTIVES AND OUTCOME MEASURES

| **Objectives** | **Outcome Measures** | **Time-point(s) of evaluation of this outcome measure (if applicable)** |
| --- | --- | --- |
| **Primary Objective** |  |  |
| To compare the effect of physical activity and aerobic exercise intervention versus usual care/minimal intervention on awake blood pressure levels in young adults of varying gestational age with elevated blood pressure | Systolic and Diastolic blood pressure measured during 24 hour ambulatory blood pressure at baseline and upon completion of 16 week intervention | Measured at baseline and upon completion of 16 week intervention |
| **Secondary Objectives** |  |  |
| To compare the sustained effect of physical activity and aerobic exercise intervention versus usual care/minimal intervention on awake blood pressure levels in young adults of varying gestational age with elevated blood pressure | Systolic and Diastolic blood pressure measured during 24 hour ambulatory blood pressure at baseline, upon completion of 16 week intervention, and at and 52 weeks post-baseline | Measured at:  - baseline  - upon completion of 16 week intervention  - 52 weeks post randomisation |
| To compare the effect of physical activity and aerobic exercise intervention versus usual care/minimal intervention on cardiopulmonary exercise performance in young adults of varying gestational age with elevated blood pressure | Oxygen uptake and carbon dioxide exchange kinetics across submaximal and peak exercise | Measured at:  - baseline  - upon completion of 16 week intervention  - 52 weeks post randomisation |
| To compare the effect of physical activity and aerobic exercise intervention versus usual care/minimal intervention on central blood pressures and vascular stiffness in young adults of varying gestational age with elevated blood pressure | - Pulse wave velocity - Augmentation index - Central blood pressure | Measured at:  - baseline  - upon completion of 16 week intervention  - 52 weeks post randomisation |
| To investigate cardiac adaptation and remodelling following exercise intervention in preterm and full term born participants compared to non-exercising controls | Cardiac Echocardiography (all participants n=200)   - Cardiac Mass - Ventricular Structure and Function - 3D-shape and functional analysis   Cardiac MRI ( MRI subgroup n=100)   - Cardiac Mass - Ventricular Structure and Function - 3D-shape and functional analysis | Echocardiography completed at:  - baseline  - upon completion of 16 week intervention  - 52 weeks post randomisation (optional)  Cardiac MRI completed at:  - baseline  - upon completion of 16 week intervention |
| To investigate the change in microvascular structure and function post exercise intervention compared to control | Retinal Imaging  Dermal Capillaroscopy | Measured at:  - baseline  - upon completion of 16 week intervention |
| To investigate the degree of natural variability in circulating angiogenic, vascular and vasoactive markers and biomarkers in blood of the healthy volunteers | Blood analysis tests including but not limited to protein and gene-expression assays | Measured at:   - Sub-study baseline visit 1 - Sub-study visit 2 (1 week post visit 1) |
| To quantify mean changes in circulating angiogenic and vascular markers and biomarkers at rest and following peak exercise between intervention and control participants | Whole, plasma, and serum blood samples at rest and following peak exercise testing | Measured at:  - baseline  - upon completion of 16 week intervention  - 52 weeks post randomisation (resting only) |
| To investigate levels of vasoactive markers and biomarkers related to renal function at rest and following peak exercise between intervention and control participants | Whole, plasma, and serum blood samples at rest and following peak exercise testing | Measured at:  - baseline  - upon completion of 16 week intervention  - 52 weeks post randomisation (resting only) |
| To investigate neurophysiological remodelling following exercise intervention compared to control | Brain MRI (MRI subgroup n=100)   - Ventricular volume and shape - Presence of white matter lesions - White matter microstructural differences including fractional anisotropy - Vessel Density and Tortuosity   Gait analysis accelerometry profile | Measured at:  - baseline  - upon completion of 16 week intervention |
| To investigate hepatic remodelling and mean change in hepatic adiposity pre and post exercise intervention compared to control | Liver MRI (MRI sub-group n=100)  Liver MRI Endpoints   - Structure and volume - Intra-hepatic lipid content - Steatohepatitis - Hepatic fibrosis | Measured at:  - baseline  - upon completion of 16 week intervention |
| To investigate the correlation between physical activity behaviour change and participants’ cognitive and psycho-social determinants of exercise including self-efficacy to exercise, motivations to exercise. | Objective measure of physical activity (7 day wear of activity monitor)  Self-reported questionnaire responses, including self-reported physical activity questionnaires, cognitive and psycho-social questionnaire items and self-efficacy measures. | Measured at:  - baseline  - upon completion of 16 week intervention  - 52 weeks post randomisation |
| **Tertiary Objectives** |  |  |
| Participant experience: assessment of individual experience following intervention | Structured interview (participants) | End of trial period |
| Economic evaluation: assessment of feasibility for scaling intervention to multi-centre trial including intervention process evaluation | EQ-5D-5L health questionnaire results  Structured interviews (study team and personnel)  Review of medical notes/GP consultations | End of trial period |

# TRIAL DESIGN

**Summary of Study**

- Design and aims

The trial is an open, parallel arm randomised control trial which will investigate the effectiveness of physical activity behaviour change and structured exercise to lower blood pressure in young adults with pre-hypertension and Stage 1 hypertension. The results will be interpreted with specific reference to preterm vs full-term birth.

- Participant profile and recruitment.

Participants will be characterised as preterm (born before 37 weeks gestation, n=100) or full term (born after 37 weeks gestation, n=100). Participants will be aged from 18 to 35 years old, with a screening blood pressure above 120mmHg systolic and/or above 80 mmHg diastolic with systolic blood pressure not exceeding 160mmHg and diastolic blood pressure not exceeding 100mmHg. Recruitment will be driven from invitation from GP records, invitation from birth hospital birth registers, open recruitment, web-based recruitment, and participation in previous studies.

In total 200 participants will be recruited and randomised following baseline measures to exercise intervention (n=100 (includes 50 preterm)) or to a comparison control group with minimal intervention (n=100 (includes 50 preterm)).

We will test the reproducibility of our blood cell and circulating biomarkers by recruiting a cohort of healthy participants aged between 18 to 35 years old (n=20). The validation study is crucial to determine the reproducibility and evaluation of experimental protocols that is required for future publications including attempt to control the variability of time factor. Participants with a clinic blood pressure below 160mmHg systolic and 100mmHg diastolic blood pressure will be eligible for inclusion. Recruitment will be driven via poster advertisement.

Study visit and measures

Participants who enter the trial will nominally complete 4 study visits within the following 6 stages of the trial:

I. Screening assessment (Visit 0)

II. Baseline (Visit 1)

III. Randomisation and allocation to intervention or control

IV. 1^st^ follow-up (Visit 2) at 16 weeks (immediately post structured exercise intervention)

V. 2^nd^ follow-up (Visit 3) - (52 weeks post randomisation)

VI. Optional exit interview reporting participant experience.

Study visits 1 & 2 will take approximately 3 hours for core study measures while visit 3 will take approximately 1.5 hours. Participants completing the MRI sub-study will complete an additional 90 minutes of MRI imaging within one week of their primary trial visit.

Core study measures will include echocardiogram for measures of cardiac structure and function, microvascular assessment with retinal imaging and dermal capillaroscopy, cardiopulmonary exercise testing (CPET) for fitness assessment, and resting and post exercise blood samples to measure inflammatory, angiogenic, vasoactive and metabolic profiles. A subgroup of study participant (n=100, 50 control and 50 intervention participants) will complete detailed MRI imaging including liver, cardiac and brain imaging.

In advance of the study visit participants will be invited to complete an online study questionnaire. The questionnaire will record correlates of physical activity behaviour and self-reported lifestyle behaviours such as smoking and alcohol consumption. Lifestyle behaviours are important determinants and risk factors for the development of elevated blood pressure and cardiovascular risk.

The study visit will conclude with participants being fitted with physical activity and 24 hour blood pressure monitors (if the baseline visit occurs within 30 days of the screening visit, a 24 hour blood pressure monitor will not be fitted at that time). Participants will be asked to wear the wrist worn activity monitors for 7 days. Participants will return monitoring devices using prepaid envelopes. If monitors are not returned after 2 weeks, the study team will give the participants a telephone call to ask for the devices to be returned. Compliance with returning self-monitoring devices in previous and ongoing ethically approved studies run by our group has been high. When the devices are returned, this will represent completion of the primary activities of the trial visit.

To test the reproducibility of the blood cell and circulating biomarkers, participants will be asked to fast for four hours prior to blood sampling but encouraged to continue drinking water. Up to 30mL of venous blood will be taken at visit 1 and again at a follow-up visit (visit 2), which will be exactly one week after visit 1. Blood samples processing, storage and experiments will be conducted as for the main TEPHRA study.

A flowchart for the planned study procedures can be found in appendix C at the end of this protocol.

# PARTICIPANT IDENTIFICATION

## Trial Participants

Individuals will be identified by gestational age as preterm or full-term born adults. The responses for potentially hypertensive individuals identified below are consistent with current (published August 2011) National institute for Health and Care Excellence (NICE) guidance for clinical management of primary hypertension in adults under 40 years old^47^.

All candidates shall have their clinic blood pressure measured at the initial screening (Visit 0). Willing candidates who have not met any exclusion criteria shall be fitted with a 24 hour blood pressure monitor to determine their 24 hour awake Ambulatory Blood Pressure (ABP). At baseline (Visit 1), study participants will have verified 24 hour awake ABP greater than 115mmHg systolic and/or greater than 75 mmHg diastolic and less than 150mmHg systolic and 95mmHg diastolic. Participants will have no therapeutic intervention for blood pressure control within the last 90 days. Hypertensive individuals will be screened for end organ hypertension damage with urine dip analysis for protein, resting ECG for left ventricular hypertrophy and fundoscopy for retinal signs of hypertension. Individuals will be excluded from the trial and referred for further assessment if hypertension end organ damage is suspected. Individuals with possible stage 1 hypertension at screening and subsequent 24 hour awake ABP > 135/85 shall be assessed and referred for further assessment as appropriate. Individuals who are referred for further assessment may be re-considered for trial participation if secondary causes of hypertension are ruled out and the individual is cleared to participate by their clinical care team. Individuals with an awake 24 hour ABP above 149/94 will be excluded from the trial and referred for follow up. The following matrix summarizes standard responses to initial screening blood pressures.

| **CLINIC BLOOD PRESSURE AT SCREENING** | **RESPONSE** |
| --- | --- |
| NORMAL |  |
| SBP < 110 **&** DBP < 70 | Complete screening & exclude from trial |
| POSSIBLE PRE-HYPERTENSION |  |
| 110 ≤ **SBP** ≤ 139 **&/or** 70 ≤ DBP ≤ 89 | Complete screening & conduct 24 hr monitor at V1 as appropriate |
| POSSIBLE STAGE I HYPERTENSION |  |
| 140 ≤ SBP ≤ 159 **&/or** ≤ 90 DBP ≤ 99 | **In all cases**: check for signs of end organ damage, if signs of end organ damage exist, exclude from trial and refer for further assessment.  **As appropriate**:  -complete screening & exclude from trial  -complete screening & refer for further assessment  -complete screening & conduct 24 hr monitor at V1 as appropriate  Note: If subsequent 24 hour awake  ABP > 135/85, consider referral for further assessment |
| POSSIBLE STAGE II HYPERTENSION |  |
| **SBP** > 159 | Exclude from trial & refer for further assessment |
| **DBP** > 99 | Exclude from trial & refer for further assessment |

**Blood Cell and Biomarker Sub-Study:**

For a small sub-cohort, participants will have their clinic blood pressure measured at the first visit. Participants with blood pressure below 160mmHg systolic and 100mmHg diastolic will be included into the sub-study (n=20) for blood cell and biomarker validation, regardless of birth history.

**Preterm-born adults**

- Born prematurely (<37 weeks completed gestation identified by self-report and when possible, birth records)

**Full-Term-born adults**

- Born at term (>37 weeks completed gestation) and with a normal birth weight for gestational age (birth weight between the 10^th^ and 90^th^ percentile for gestational age, birth weight identified by self-report and when possible, birth records).

Prior to study inclusion, the following means will be used to verify participant birth history:

- - Birth history verified in a previous study
  - Verification of birth history by review of birth/medical records (eg birth ledger, digital record, personal child health record or other valid documented source)
  - Corroboration via high confidence report of family member or guardian

If birth history cannot be verified, the candidate shall be excluded from the trial.

Participants will be adults from 18-35 years old.

Participants will be randomised using stratification criteria of age, sex, and gestational age.

## Inclusion Criteria (all participant apart from Validation sub-study)

- Participant is willing and able to give informed consent for participation in the study.
- Male or Female, from 18 to 35 years old.
- Verified birth history: preterm birth (<37 weeks) or full-term birth (>37 weeks)
- Ability to access and use computer/internet
- Willing to complete duration of intervention, follow-up and attend study visits at the John Radcliffe Hospital (participants may still withdraw at any time without providing an explanation or rationale)
- 24 hour awake ABP greater than 115/75 mmHg
- Able (in the investigator's opinion) and willing to comply with all study requirements.

## Exclusion Criteria (all participant apart from Validation sub-study)

The participant may not enter the study if ANY of the following apply:

- Clinic blood pressure greater than 159mmHg systolic and/or 99mmHg diastolic at initial screening
- Pregnancy
- 24 hour awake ABP greater than 150mmHg systolic and/or 95mmHg diastolic
- Clinic blood pressure greater than 140mmHg systolic and/or 90 mmHg diastolic plus evidence of end organ damage secondary to hypertension
- Simultaneous participation in another human or clinical randomized trial (if there is any possibility of compromising health, safety, or well-being, or any possible compromise of study data)
- Unable to walk briskly on the flat for 15 minutes
- Those currently maintaining levels of cardiovascular fitness and activity at or above the levels required for the intervention arm
- Unable to attend the regular supervised exercise sessions
- Use of beta-blockers such as atenolol or equivalent
- BMI >35 kg/m^2^
- Major contra-indications to exercise participation
- Evidence of cardiomyopathy
- Evidence of inherited cardiac conduction abnormalities
- Evidence of congenital heart disease or significant chronic disease relevant to cardiovascular status

**The following are exclusion criteria for the MRI sub-study only:**

| - a permanent pacemaker | - shrapnel injuries |
| --- | --- |
| - metal clips in blood vessels of the brain | - other metal or electronic implants affected by the magnetic field - Unsuitable for MRI based on responses on the MRI safety screening form |
| - an injury to the eye involving fragments of metal |  |

## Inclusion criteria for TEPHRA blood validation sub-study

- Healthy participant that is willing and able to give informed consent for participation in the study.
- Male or female, from 18 to 35 years old.
- Verified birth history: preterm birth (<37 weeks) or full-term birth (>37 weeks)
- Clinic blood pressure below 160/100 mmHg
- Able (in the investigator's opinion) and willing to comply with all study requirements.

## Exclusion criteria for TEPHRA blood validation sub-study

The participant may not enter the study if ANY of the following apply:

- Pregnancy
- Use of beta-blockers such as atenolol or equivalent
- BMI >35 kg/m^2^
- Evidence of cardiomyopathy, inherited cardiac conduction abnormalities, congenital heart disease or significant chronic disease relevant to cardiovascular status

# TRIAL PROCEDURES

## Recruitment

**Participant Identification and approach**

**Local Practice Recruitment**

Collaborating primary care practices will screen practice databases for adults aged of 18 to 35 years old with a documented clinic blood pressure above 125/80 mmHg in the last 24 months. The direct care teams from the respective practices will then send a letter of invitation to the identified individuals. The letter will ask individuals interested in taking part to contact the study team directly via study email or telephone.

Potential candidates will also be recruited through the Outpatient Hypertension Clinic at the John Radcliffe hospital. Referrals to the outpatient service made over the last 5 years will be reviewed by the clinical team to screen for eligible participants. Potential participants will then be sent an invitation letter signed by the lead physician from the service along with a study information sheet.

**Open Advertising**

Participants will be recruited through open advertising in the local community. Advertising will be facilitated through offering elective blood pressure information and screening stations where trial information will be available for those interested, and by placing posters in the Oxford area. Primary advertising locations will include: Oxford University Hospitals NHS Foundation Trust, University of Oxford Colleges and the Oxford Brookes University. Posters will be placed on general and research department notice boards whilst blood pressure screening and information stations will be set up in common areas that do not block foot traffic. University email bulletins will be approached to share an invitation email with their readership. For those who express interest at a blood pressure screening station, the individual will be asked if they would be happy to provide their contact information and receive a follow—up phone call or email (their preference) in 1-2 weeks. All interested individuals will be encouraged to contact the study team via email or telephone for further information. As described below, targeted open advertising will be accomplished via: internet (web-based), recruitment talks, partnering with local charities, and local media.

Web-Based Advertising

There will be two arms of web-based advertising. The first arm will include adverts placed on web pages for: Radcliffe Department of Medicine, Oxford University Hospitals, Brookes University, and Oxford University and Colleges. The second arm of web-based advertising will be procured through TrialSpark, an Oxford-based company that conducts clinical trial recruitment and has an established record of ensuring all ethical and data protection standards are fully maintained. TrialSpark will run approved, on-line study adverts that are optimized to reach potential study participants. Interested individuals will be able to access a website with additional study details and will be asked to contact the study team directly via email or telephone for further information. Adverts may be shown on google, facebook, twitter, linkedin, pinterest or other similar websites.

Recruitment talks at professional societies/organisations

The research team will monitor meetings and events for well-respected professional societies operating in Oxfordshire. The team may contact a society to see if they would be happy to allow the research team to offer blood pressure screening or a brief talk about the study at a society event or meeting. Those attending who express interest will be offered a study leaflet and asked if they would be happy to provide their contact information and receive a follow—up phone call or email (their preference) in 1-2 weeks. All interested individuals will be encouraged to contact the study team via email or telephone for further information.

Media advertising

Local Television and Radio will be approached with support of the Oxford University Hospitals NHS Foundation Trust Communications department to advertise the study. Interested individuals will be asked to contact the study team via email or telephone for further information.

Charitable Advertising

Study adverts will be placed in the newsletters and on the social media sites of the local preterm charity SNNAP, and the national preterm charity Bliss.

In collaboration with the British Heart Foundation or other well-respected local charities, advertising will be accomplished via research team members conducting blood pressure screening and information stations, and by placing posters at charity locations in Oxfordshire. For those interested at blood pressure screenings, they will be provided with a study participant information leaflet and will be asked if they would be happy to provide their contact information and receive a follow—up phone call or email (their preference) in 1-2 weeks. All interested individuals will be encouraged to contact the study team via email or telephone for further information.

**Birth Records**

Ethical approval has previously been provided to invite individuals born within the Oxford University Hospitals Trust to take part in the both the Exercise and Blood Pressure Study Oxfordshire (Ref 14/SC/0281) study and also the Young Adult Cardiovascular Health 2 study (Ref 14/SC/0275). Individuals who have returned up to date contact details and have consented to be contacted in the future will be forwarded a study invitation letter. Following initial invitation, those interested in participating in the current study will be asked to contact the study team via email or telephone for further information.

Recruitment from birth registers will be expanded to include neonatal care facilities provided by Buckinghamshire Healthcare NHS Trust (Stoke Mandeville Hospital), Great Western Hospitals NHS Trust (Swindon) and Royal Berkshire NHS Foundation Trust (Reading). Potential participants will be identified by their treating healthcare services. The direct care teams from the respective healthcare services, supported by the local NIHR research network, will send a letter of invitation to the identified individuals. The letter will ask individuals interested in taking part to contact the study team directly via study email or telephone.

**Previous study participants**

Participants from previous ethically approved studies who expressed interest to participate in future studies and consented to being contacted will be invited to consider participation. Participants in established cohorts will be approached through the lead investigators of these studies. Individuals will be contacted with an initial email or letter which will be followed up after two weeks with a telephone call.

**Response to expressions of interest from potential participants**

Where persons express an interest in the study, the initial contact is followed up (according to the person’s wishes) by a telephone call, further letter, e-mail or, by meeting the person. They will be provided with the Participant Information Leaflet which will be sent with an accompanying cover letter. Following provision of the Participant Information Leaflet, individuals will be contacted after a minimum of 48 hours and given an opportunity to talk through any issues. The study investigator will answer any questions from the potential participant relating to the study. The study investigator will then request verbal agreement to discuss study inclusion and exclusion criteria. The conversation will be documented on a pseudo-anonymised screening log which will be stored separately from the potential participant’s details. Following confirmation of eligibility, potential participants will be invited to complete the screening visit.

## Informed Consent

Eligible individuals willing to participate in the study will be provided with the full Participant Information Leaflet detailing the study information in advance of the informed consent process. Only after participants have had a minimum of 24 hours to review the study information will they be invited to schedule a time for their screening visit. The informed consent process will be completed on the day of the screening study visit.

The participant must personally sign and date the latest approved version of the Informed Consent form before any study specific procedures are performed.

Written and verbal versions of the Participant Information Leaflet and Informed Consent will be presented to the participants detailing no less than: the exact nature of the study; what it will involve for the participant; the implications and constraints of the protocol; the known side effects and any risks involved in taking part. It will be clearly stated that the participant is free to withdraw from the study at any time for any reason without prejudice to future care, and with no obligation to give the reason for withdrawal.

The participant will be allowed as much time as wished to consider the information and full opportunity to question the investigator, their GP or other independent parties to decide whether they will participate in the study. Written informed consent will then be obtained by means of participant dated signature and dated signature of the person who presented and obtained the informed consent. The person who obtained the consent must be suitably qualified and experienced, and have been authorised to do so by the Chief/Principal Investigator. A copy of the Informed Consent form will be given to the participant. The original signed form will be retained at the study site.

## Screening and Eligibility Assessment

**Visit 0 Screening & Eligibility Assessment (30 min)**

Candidate screening will be completed post consent. If an exclusionary item is verified during the screening, the investigator will notify the candidate, not perform additional screening actions and will refer for follow-up if appropriate and the candidate consents.

**Consent**

Candidates will be asked for their consent to access their medical records including birth and neonatal records. In absence of obstetric records, self-reported birth history will be relied on. If possible, participants will be asked to confirm their birth and pregnancy details with their parents.

Personal data as follows will be collected and stored separately. This is to facilitate contact with participants during the study, and to arrange baseline and follow-up visits:

- Forename and surname
- Address including postcode
- Date of Birth
- Medical record number (to allow access to patient’s medical records)
- Telephone number
- Email address

**Questionnaire**

A screening questionnaire will request the following information:

- Sex, age at entry to the study, birth history (if known)
- Reported pregnancy status
- Use of recreational substances to identify exclusionary recreational drug use
- Medical history to identify exclusionary medical conditions or pharmaceutical usage
- Participation in other trials
- Self-reported current physical activity levels
- Willingness to change physical activity and ability to access and attend supervised exercise sessions for 16 continuous weeks

**Physical Screening**

Anthropometric measurements including body composition measures, height, weight, BMI, waist to hip ratio, and resting blood pressure shall be collected. Participants will have their blood pressure checked after 5 minutes rest using the automated mode of a sphygmomanometer. 3 blood pressure readings will be taken at intervals of 1 minute. For the outcome measure of blood pressure control the mean of the second and third readings will be used. Screening blood pressure will be assessed in accordance with the candidate screening blood pressure and response identified in section 7.1.

A clinically trained study investigator, with an honorary clinical contract with the Oxford University Hospitals Trust will review questionnaire responses and eligibility for study participation. The study investigator will clarify if there are significant contraindications, diseases or disorders which, in the opinion of the investigator, might influence the individual’s ability to participate in the study. If participants have clinic blood pressure above 140 mmHg systolic and 90 mmHg diastolic they will be invited to be screened for evidence of end organ damage. Participants with elevated blood pressure and signs of end organ damage will be excluded from trial participation and referred for appropriate clinical follow-up. Participants with blood pressure from 140/90 to 159/99 mmHg and no evidence of end organ damage will be allowed to move forward in the study. Blood pressure measurements will be assessed in accordance with the screening blood pressure and response identified in section 7.1.

## Randomisation

Participants meeting the main study inclusion criteria will be randomly allocated 1:1 to the two treatment arms. This will be undertaken using a computerised randomisation program. A minimisation algorithm (with a random element) will be used to ensure balanced allocation across treatment groups for key prognostic factors: gender, age and gestational age of participants. The strata for each prognostic factor are as follows:

∙ Gender: Male/Female;

∙ Age: <24 years old, 24-29 years old, 30-35 years old;

∙ Gestational age: ≤32 weeks, 32-37 weeks, > 37 weeks.

Allocation will be concealed using an online randomization program following completion of baseline study measures. Due to the nature of the intervention participants will not be blinded. To reduce potential bias, primary and secondary outcome data will be analysed remote from the intervention by operators blinded to participant identifier with results verified by a second blinded research team member.

An emergency back-up randomization schedule will be prepared in advance and stored for use when the online system in unavailable.

Randomisation will be completed with support from the Centre for Statistics in Medicine using a secure web-based randomisation service.

## Baseline Assessments

### Visit 1 (approx. 3 hours)

Participants will be asked to attend the Oxford Cardiovascular Clinical Research Facility, having consumed only water for 4 hours beforehand.

### Core Study Measures (n=200)

### Lifestyle and physical activity questionnaire (30 min)

Study visit participants will be asked to complete the study questionnaire by hard-copy or a secure computer-based platform. Computer based questionnaires will be link-anonymised using unique study codes.

The questionnaire combines validated questions piloted or used in previous studies. Information will be collected on smoking frequency and alcohol consumption. Physical activity questionnaires will provide a self-report measure of activity (Recreational Physical Activity Questionnaire (RPAQ)) and determinants of activity including environmental perceptions. Questionnaire items will also be used to provide a measure of self-efficacy, enjoyment and motivation to engage with physical activity. The questionnaire includes measures of health-related quality of life captured through the SF-8 Health Survey question elements and the standardised Eq-5D-5L quality of life questionnaire. These questionnaire elements have been combined into a single study visit questionnaire which takes approximately 30 minutes to complete. As appropriate, participants may be sent a hard-copy questionnaire by post or an email prompt in advance of the study visit. Completion will be checked during the baseline visit. If required, participants will be provided with an opportunity to complete the questionnaire during the visit.

### Physical Examination and ECG (15 mins)

(Note: If baseline visit is within 30 days of the screening visit, anthropometric data from screening shall be used) Assessments including body composition measures, height, weight, BMI, waist to hip ratio, gait analysis, and resting blood pressure will be taken. Participants will undergo a brief gait analysis by completing a monitored 10 meter walk while wearing an accelerometer fastened to their low back. Participants will have their blood pressure checked after 5 minutes rest using the automated mode of a sphygmomanometer. 3 blood pressure readings will be taken at intervals of 1 minute. The first reading will be discarded and not used toward the mean blood pressure calculated for the visit.

A 12 lead ECG shall be conducted and reviewed by a clinically trained study investigator with an honorary contract with the Oxford University Hospitals Trust. If the investigator determines that ECG results show evidence of exclusionary cardiac disease, the candidate will be excluded from the trial and referred for appropriate clinical follow up. Candidates who are referred for follow-up may be considered for participation at a later date if fully cleared by their clinical care team after formal clinical assessment. The ECG recording may be accomplished as a stand-alone measure or as part of other study measure procedures which use ECG monitoring such as cardiopulmonary exercise testing.

**Vascular Measures and Central Blood Pressures (5 mins)**

Resting measures of vascular stiffness, pulse wave velocity and central blood pressure will be collected using non-invasive devices.

**Microvascular Assessment (25 mins)**

Retinal photography will be completed using a digital camera and imaging soft-ware following an established protocol. Imaging is non-invasive with no requirement for topical drops and does not require removal of contact lenses.

Capillary density will be measured using a microscope on the dorsal surface of the finger.

**Echocardiogram cardiac ultrasound scan (25 mins)**

Cardiac ultrasound imaging will be used to evaluate cardiac structure and function. Cardiac ultrasound will be completed by an operator trained in echocardiography.

### Cannulation and Blood Sampling (10 mins)

A venous blood sample (approximately 50mls) will be taken at rest for a) whole blood, plasma and serum lipid and inflammatory marker analysis, b) analysis of biochemistry and metabolism and c) analysis of biomarkers associated with inflammation, angiogenesis and endothelial activation.

Further venous blood samples (approximately 100mls total) will be taken during and after exercise to repeat the above tests and measure changes in metabolism associated with exercise. To ensure the cannula does not become blocked the cannula may need to be flushed with 5mls of normal saline after each sample is taken. This would equate to up to a total of 20mls of normal saline being used to maintain the patency of the cannula. This a safe method used in clinical practice. This will allow collection of blood without the need for multiple needle pricks and is intended to improve the experience of the participant.

Blood samples will be centrifuged and plasma, serum, cells and DNA stored. All samples will be retained in a secure environment for future analysis and will be stored in an anonymous format at the John Radcliffe Hospital and Wellcome Trust Centre for Human Genetics, University of Oxford under the custodianship of Professor Paul Leeson, Cardiovascular Clinical Research Facility, Division of Cardiovascular Medicine. The samples of serum and DNA will be stored for 10 years and may be used in future ethically approved studies as our understanding of blood vessel function grows. All other human materials will be disposed of at the end of the study in accordance with the Human Tissue Authority Code of Practice.

**Cardiopulmonary Exercise Testing (40 mins)**

Cardiac function and oxygen requirements in response to incremental increase in workload will be measured via cardiopulmonary exercise test. The exercise protocol is a validated incremental protocol with established use in clinical and research practice. The exercise protocol is currently utilised in ongoing ethically approved studies conducted by the Division of Cardiovascular Medicine, and is performed on a stationary bike. The test commences with resting measures of spirometry.

During the exercise testing we will measure the oxygen uptake of the exercising thigh muscles. This is recorded using near-infrared spectroscopy which measures the refraction of light from circulating haemoglobin to measure the oxygen concentration. The device measures the change in oxygen concentration to provide a measure of the muscle oxygen uptake. It is a non-invasive, painless technique with no associated risks. To make this measurement we will attach two sticky sensors to the thigh muscle on one of the participants’ legs.

Participants will exercise with an incrementally increasing workload (increasing resistance to pedal against) to assess peak exercise capacity, the test will last 10 to 20 minutes. During the test, heart rate will be recorded using ECG monitoring, blood pressure will be measured at intervals and participants will report effort using the Borg exertion scale.

**Fitting monitoring devices (5 mins)**

### 24 hour Ambulatory Blood Pressure (ABP)

This will be properly fitted to the participant at the end of the visit. It consists of a blood pressure cuff worn on the upper arm that will fit under loose clothing. The cuff is attached by a lead to a small box which is similar in size to a Walkman. This can be placed in a pocket or attached to a belt which the study team will provide. This portable device will measure blood pressures half hourly during the day and hourly at night. ADP data will be verified by a trained study investigator prior to randomisation.

### 7 day accelerometer

This will be attached to the participant at the end of the study visit. It consists of a wrist worn accelerometer similar in design to a wrist worn watch. Wrist worn accelerometers have high compliance and reliability and are validated measures of physical activity. Participants will be asked to wear the accelerometer for 7 days.

Stamped addressed envelopes will be provided for the return of monitoring devices.

**MRI Subgroup Measures**

Subgroup [n=100, including up to 50 preterm born participants and up to 50 full term born participants]

Participants in the MRI subgroup will complete MRI imaging of the Brain, Liver and Heart. Imaging will be completed at the Oxford Centre of Magnetic Resonance. For participants in this subgroup, study visits will commence with image acquisition.

**MRI Brain (30 mins)**

MRI imaging will be used to quantify brain structure and volume on a 1.5T – 7T scanner once the participant has been deemed to be scanner safe, following an established protocol.

**Cardiovascular Magnetic Resonance** **(CMR) (30 mins)**

MRI imaging will be used to quantify cardiovascular structure and volume on a 1.5T – 7T scanner once the participant has been deemed to be scanner safe, following an established protocol.

**MRI Liver Imaging (10 mins)**

A liver magnetic resonance scan will be performed at the same sitting as the cardiac magnetic resonance.

Depending on operator and facility availability, the MRI imaging will be scheduled within 5 days of completing the core study measures.

## Subsequent Visits

**Visit 2 & Visit 3 - Follow-up of Primary and Secondary Outcome Measures**

Participants will be followed up at 16 weeks +/- 1 week (Visit 2) and 52 weeks +/- 2 weeks (Visit 3) post randomisation and commencing the respective study arms. Each visit will commence with the study team confirming the participant’s consent to take part.

Study visit 2 will replicate baseline study measures including core study measures and MRI imaging for MRI subgroup. Study visit 3 will include only: questionnaire, physical examination (without 10m walk), echocardiogram (optinal), resting blood sampling, cardiopulmonary exercise testing, and fitting of monitoring devices.

### Visit 2 (approx. 3 hours)

Participants will be asked to attend the Oxford Cardiovascular Clinical Research Facility, having consumed only water for 4 hours beforehand.

### Core Study Measures (n=200)

### Lifestyle and physical activity questionnaire (30 min)

Study visit participants will be asked to complete the study questionnaire using via hard-copy or a secure computer-based platform.

The questionnaire combines validated questions piloted or used in previous studies. Information will be collected on smoking frequency and alcohol consumption. Physical activity questionnaires will provide a self-report measure of activity (Recreational Physical Activity Questionnaire (RPAQ)) and determinants of activity including environmental perceptions. Questionnaire items will also be used to provide a measure of self-efficacy, enjoyment and motivation to engage with physical activity. The questionnaire includes measures of health related quality of life captured through the SF-8 Health Survey question elements and the standardised Eq-5D-5L quality of life questionnaire. These questionnaire elements have been combined into a single study visit questionnaire which takes approximately 30 minutes to complete. As appropriate, participants may be sent a hard-copy questionnaire by post or an email prompt in advance of the study visit. Completion will be checked during the baseline visit. If required, participants will be provided with an opportunity to complete the questionnaire during the visit.

### Physical Examination (15 mins)

Assessments including body composition measures, height, weight, BMI, waist to hip ratio, gait analysis, and resting blood pressure will be taken. Participants will undergo a brief gait analysis by completing a monitored 10 meter walk while wearing an accelerometer fastened to their low back. Participants will have their blood pressure checked after 5 minutes rest using the automated mode of a sphygmomanometer. 3 blood pressure readings will be taken at intervals of 1 minute. The first reading will be discarded and not used toward the mean blood pressure calculated for the visit.

**Microvascular Assessment (25 mins)**

Retinal photography will be completed using a digital camera and imaging soft-ware following an established protocol. Imaging is non-invasive with no requirement for topical drops and does not require removal of contact lenses.

Capillary density will be measured using a microscope on the dorsal surface of the finger.

**Echocardiogram cardiac ultrasound scan (25 mins)**

Cardiac ultrasound imaging will be used to evaluate cardiac structure and function. Cardiac ultrasound will be completed by an operator trained in echocardiography.

### Cannulation and Blood Sampling (10 mins)

A venous blood sample (approximately 50mls) will be taken at rest for a) whole blood, plasma and serum lipid and inflammatory marker analysis, b) analysis of biochemistry and metabolism and c) analysis of biomarkers associated with inflammation, angiogenesis and endothelial activation.

Further venous blood samples (approximately 100mls total) will be taken during and after exercise to repeat the above tests and measure changes in metabolism associated with exercise. To ensure the cannula does not become blocked the cannula may need to be flushed with 5mls of normal saline after each sample is taken. This would equate to up to a total of 20mls of normal saline being used to maintain the patency of the cannula. This a safe method used in clinical practice. This will allow collection of blood without the need for multiple needle pricks and is intended to improve the experience of the participant.

Blood samples will be centrifuged and plasma, serum, cells and DNA stored. All samples will be retained in a secure environment for future analysis and will be stored in a link-anonymised format at the University of Oxford Cardiovascular Clinical Research Facility and Wellcome Trust Centre for Human Genetics, University of Oxford under the custodianship of the Division of Cardiovascular Medicine. The samples of serum and DNA will be stored for at least 10 years and may be used in future ethically approved studies as our understanding of blood vessel function grows. All other human materials will be disposed of at the end of the study in accordance with the Human Tissue Authority Code of Practice.

**Cardiopulmonary Exercise Testing (40 mins)**

Cardiac function and oxygen requirements in response to incremental increase in workload will be measured via cardiopulmonary exercise test. The exercise protocol is a validated incremental protocol with established use in clinical and research practice. The exercise protocol is currently utilised in ongoing ethically approved studies conducted by the Division of Cardiovascular Medicine, and is performed on a stationary bike. The test commences with resting measures of spirometry.

During the exercise testing we will measure the oxygen uptake of the exercising thigh muscles. This is recorded using near-infrared spectroscopy which measures the refraction of light from circulating haemoglobin to measure the oxygen concentration. The device measures the change in oxygen concentration to provide a measure of the muscle oxygen uptake. It is a non-invasive, painless technique with no associated risks. To make this measurement we will attach two sticky sensors to the thigh muscle on one of the participants’ legs.

Participants will exercise with an incrementally increasing workload (increasing resistance to pedal against) to assess peak exercise capacity, the test will last 10 to 20 minutes. During the test, heart rate will be recorded using ECG monitoring, blood pressure will be measured at intervals and participants will report effort using the Borg exertion scale.

**Fitting monitoring devices (5 mins)**

### 24 hour Ambulatory Blood Pressure

This will be attached to the candidate at the end of the visit. It consists of a blood pressure cuff worn on the upper arm that will fit under loose clothing. The cuff is attached by a lead to a small box which is slightly larger than a deck of cards. This can be placed in a pocket or attached to a belt which the study team will provide. This portable device will measure blood pressures half hourly during the day and hourly at night. A stamped addressed envelope will be provided to return the device after use.

### 7 day accelerometer

This will be attached to the participant at the end of the study visit. It consists of a wrist worn accelerometer similar in design to a wrist worn watch. Wrist worn accelerometers have high compliance and reliability and are validated measures of physical activity. Participants will be asked to wear the accelerometer for 7 days.

Stamped addressed envelopes will be provided to return devices.

**MRI Subgroup Measures** Participants will be asked for their consent to have images stored at the time of initial consent. All MR will be performed on a 1.5Tesla and/or 3.0Tesla MR scanner.

**Brain MRI (30 mins)**

MRI imaging will be used to quantify brain structure and volume once the participant has been deemed to be scanner safe, following an established protocol. Participants will lie in a supine position with a dedicated coil utilised for image optimization.

**Cardiovascular Magnetic Resonance** **(CMR) (30 mins)**

MRI imaging will be used to quantify cardiovascular structure and function once the participant is deemed scanner safe. All participants will undergo a standardised CMR protocol. Participants will lie in a supine position and the MRI operator shall place a dedicated cardiac coil around their chest. Images are obtained using breath hold and ECG gating.

**MRI Liver Imaging (10 mins)**

A liver magnetic resonance scan will be performed at the same sitting as the brain and cardiac magnetic resonance.

Depending on operator and facility availability, the MRI imaging will be accomplished within 5 days of completing the core study measures.

### Visit 3 (approx. 1.5 hours)

Participants will be asked to attend the Oxford Cardiovascular Clinical Research Facility, having consumed only water for 4 hours beforehand.

### Core Study Measures (n=200)

### Lifestyle and physical activity questionnaire (30 min)

Study visit participants will be asked to complete the study questionnaire using via hard-copy or a secure computer-based platform.

The questionnaire combines validated questions piloted or used in previous studies. Information will be collected on smoking frequency and alcohol consumption. Physical activity questionnaires will provide a self-report measure of activity (Recreational Physical Activity Questionnaire (RPAQ)) and determinants of activity including environmental perceptions. Questionnaire items will also be used to provide a measure of self-efficacy, enjoyment and motivation to engage with physical activity. The questionnaire includes measures of health related quality of life captured through the SF-8 Health Survey question elements and the standardised Eq-5D-5L quality of life questionnaire. These questionnaire elements have been combined into a single study visit questionnaire which takes approximately 30 minutes to complete. As appropriate, participants may be sent a hard-copy questionnaire by post or an email prompt in advance of the study visit. Completion will be checked during the baseline visit. If required, participants will be provided with an opportunity to complete the questionnaire during the visit.

### Physical Examination (15 mins)

Assessments including body composition measures, height, weight, BMI, waist to hip ratio and resting blood pressure will be taken. Participants will have their blood pressure checked after 5 minutes rest using the automated mode of a sphygmomanometer. 3 blood pressure readings will be taken at intervals of 1 minute. The first reading will be discarded and not used toward the mean blood pressure calculated for the visit.

**Echocardiogram cardiac ultrasound scan (Optional) (25 mins)**

Cardiac ultrasound imaging will be used to evaluate cardiac structure and function. Cardiac ultrasound will be completed by an operator trained in echocardiography. This scan is optional at visit 3.

### Blood Sampling (10 mins)

A venous blood sample (approximately 50mls) will be taken at rest for a) whole blood, plasma and serum lipid and inflammatory marker analysis, b) analysis of biochemistry and metabolism and c) analysis of biomarkers associated with inflammation, angiogenesis and endothelial activation.

Blood samples will be centrifuged and plasma, serum, cells and DNA stored. All samples will be retained in a secure environment for future analysis and will be stored in a link-anonymised format at the University of Oxford Cardiovascular Clinical Research Facility and Wellcome Trust Centre for Human Genetics, University of Oxford under the custodianship of the Division of Cardiovascular Medicine. The samples of serum and DNA will be stored for at least 10 years and may be used in future ethically approved studies as our understanding of blood vessel function grows. All other human materials will be disposed of at the end of the study in accordance with the Human Tissue Authority Code of Practice.

**Cardiopulmonary Exercise Testing (40 mins)**

Cardiac function and oxygen requirements in response to incremental increase in workload will be measured via cardiopulmonary exercise test. The exercise protocol is a validated incremental protocol with established use in clinical and research practice. The exercise protocol is currently utilised in ongoing ethically approved studies conducted by the Division of Cardiovascular Medicine, and is performed on a stationary bike. The test commences with resting measures of spirometry.

During the exercise testing we will measure the oxygen uptake of the exercising thigh muscles. This is recorded using near-infrared spectroscopy which measures the refraction of light from circulating haemoglobin to measure the oxygen concentration. The device measures the change in oxygen concentration to provide a measure of the muscle oxygen uptake. It is a non-invasive, painless technique with no associated risks. To make this measurement we will attach two sticky sensors to the thigh muscle on one of the participants’ legs.

Participants will exercise with an incrementally increasing workload (increasing resistance to pedal against) to assess peak exercise capacity, the test will last 10 to 20 minutes. During the test, heart rate will be recorded using ECG monitoring, blood pressure will be measured at intervals and participants will report effort using the Borg exertion scale.

**Fitting monitoring devices (5 mins)**

### 24 hour Ambulatory Blood Pressure

This will be attached to the candidate at the end of the visit. It consists of a blood pressure cuff worn on the upper arm that will fit under loose clothing. The cuff is attached by a lead to a small box which is slightly larger than a deck of cards. This can be placed in a pocket or attached to a belt which the study team will provide. This portable device will measure blood pressures half hourly during the day and hourly at night. A stamped addressed envelope will be provided to return the device after use.

### 7 day accelerometer

This will be attached to the participant at the end of the study visit. It consists of a wrist worn accelerometer similar in design to a wrist worn watch. Wrist worn accelerometers have high compliance and reliability and are validated measures of physical activity. Participants will be asked to wear the accelerometer for 7 days.

Stamped addressed envelopes will be provided to return devices.

### Visit 4. Exit Interview

We will conduct structured interviews with participants in both arms who consent to participate in the optional exit interview to assess their experience of study participation and to identify and explore factors related to the successful (or unsuccessful) implementation of the intervention. This will provide data regarding the views of participants as to the acceptability of supervised exercise and self-monitored physical activity behavior change.

Participants in both arms will be interviewed to explore barriers and facilitators to supervised exercise, self-monitoring physical activity and trial participation. The control group will be interviewed to allow comparison of patient experience in the two arms.

The structured interviews will take approximately 10-15 minutes. There will be 5 questions for both groups, and an additional 5 questions in the intervention group.

An important focus of the interviews will be on identifying: perceived barriers; where similarities and differences in perception lie; appropriate behaviors and information sharing between participant and intervention team; and participant satisfaction. We will use a structured interview approach with free text options, as this provides a reproducible format for consistency across interviews, while also allowing for further (free text) explanation. Structured responses will be entered on to a pre-coded scoring sheet. Free text responses will be audio-recorded and transcribed in order to capture all participant experiences.

All participants being enrolled into the trial will be asked if they consent to participate in this optional exit interview. The interview will be conducted by a researcher independent to the intervention delivery team. Participants will be given the option of a face to face interview at the end of study visit 4 or a telephone interview at the participants’ convenience.

## Sub-Study Visits: Circulating biomarker validation and evaluation visit 1 and visit 2

### Recruitment of participants for TEPHRA blood validation sub-study

A sub-cohort of healthy individuals will be invited via poster advertisement with primary advertising locations including: Oxford University Hospitals NHS Foundation Trust, University of Oxford Colleges and the Oxford Brookes University, to participate in a sub-study for blood cell and circulating biomarker evaluation.

The additional cohort of participants (n=20) will be invited to the blood cell and circulating biomarker validation and evaluation study. Participants will be asked to attend the Oxford Cardiovascular Clinical Research Facility at the John Radcliffe Hospital in Oxford having consumed only water for 4 hours beforehand.

### Visit 1

### Consent (10 mins)

Participants will be asked for their consent to take part in the sub-study. Personal data as follows will be collected and stored separately. This is to facilitate contact with participants during the study, and to arrange baseline and follow-up visits:

- Forename and surname
- Address including postcode
- Date of Birth
- Telephone number
- Email address

Participant examination will be completed post-informed consent. If an exclusionary item is verified during the visit, the investigator will notify the participant. No additional research procedures will be performed, and, with the participant’s consent, the participant will be referred for clinical follow-up.

### Eligibility screening (5 mins)

Participants will have their blood pressure checked after 5 minutes rest using the automated mode of a sphygmomanometer. Three blood pressure readings will be taken at intervals of 1 minute. Clinic blood pressure above 159/99 mmHg will be excluded and no additional procedure will be performed.

### Study procedures

### Physical examination (10-15 mins)

Anthropometric measurements including height, weight and bio-impedance measurements will be collected. Participants will have their blood pressure taken again for the second visit. Participants will have their blood pressure checked after 5 minutes rest using the automated mode of a sphygmomanometer. Three blood pressure readings will be taken at intervals of 1 minute.

### Blood sampling (10 mins)

If a participant is eligible, a venous fasting blood sample (approximately 30mls) will be taken at rest for a) whole blood, plasma and serum lipid and inflammatory marker analysis, b) analysis of biochemistry and metabolism c) analysis of biomarkers associated with inflammation, angiogenesis and endothelial activation.

Blood samples will be centrifuged, and plasma, serum, cells and DNA stored. Samples collected will be used for several experiments including looking at genes and protein expression to ensure the reproducibility and validity between the samples collected from the same individual from two independent visits. All samples will be retained in a secure environment for future analysis which will be performed in batches. Samples will be stored in an anonymous format in the Cardiovascular Clinical Research Facility, Division of Cardiovascular Medicine, University of Oxford at the John Radcliffe Hospital site and Wellcome Centre for Human Genetics under the custodianship of Professor Paul Leeson. Relevant Human Tissues samples will be destroyed at the end of study unless consent has been obtained from the participant to retain their samples for future research. In this case, samples will be transferred to another ethically approved study and a copy of the consent will be kept. Non-relevant Human Tissues samples will be kept indefinitely.

### Visit 2

Participants will be invited back exactly one week later for the follow-up visit (visit 2). Study procedures will be as per listed under visit 1.

## Additional sample collection and handling

The sample collection and handling are described elsewhere in the protocol. Upon the completion of the studies, samples from the YACHT 2 Study (14/SC/0275) and Expresso (14/SC/0281) will be transferred to this study should there be consent from the participant to use samples in future ethically approved studies in order to assess blood biomarkers. Copies of the consent forms will be retained with the samples.

## Discontinuation/Withdrawal of Participants from Trial

Each participant has the right to withdraw from the trial at any time. In addition, the Investigator may discontinue a participant from the study at any time if the Investigator considers it necessary for any reason including:

- Pregnancy
- Ineligibility (either arising during the study or retrospectively having been overlooked at screening)
- Significant deviation from the study protocol
- Withdrawal of Consent
- Loss to follow up
- Contraindication to exercise participation

If participants withdraw from the study the study investigators will ask the participants if they can make use of the information that has been collected up to the time of withdrawal to facilitate intention to treat analysis. Participants may withdraw consent for any use of their samples or data at any time. If this is the case we will destroy any identifiable samples or information we hold about the participant. Participants do not need to provide any reason for withdrawal; should they freely offer a reason it will be recorded in the CRF/eCRF. The research team may recruit additional participants to replace participants who have withdrawn.

## Definition of End of Study

The end of study is the date of the last visit or telephone interview if completing an optional exit telephone exit interview of the last participant.

# INTERVENTIONS

# Supervised Aerobic Exercise and Self-Monitoring Physical Activity Intervention

# (See Appendix E)

**Supervised Exercise**

Participants in the intervention arm will complete 16 weeks training with weekly supervised aerobic interval sessions, 3 sessions per week, duration 40-60 minutes, plus 15 minutes warm-up and cool-down (total duration 70-90 minutes). Participants will be provided with a 4 month gym membership, training will be co-ordinated by the study team in collaboration with the team from the Movement Science Department at the Oxford Brookes University. Gym membership will be secured at facilities local to the participant and pre-approved by the study team. Attendance and progress during the supervised intervention will be monitored weekly by the study team.

Exercise prescription will be based on 60-80% maximal aerobic capacity defined using heart rate response during peak cardiopulmonary exercise testing at baseline. Heart rate monitors will be used to maintain intensities of sessions. The supervised intervention replicates similar strategies identified during systematic review of the randomised control literature delivering exercise intervention for blood pressure reduction^48,49^. The minimum dose to start effecting positive changes in cardiovascular fitness is estimated to be 40 minutes of moderate intensity sessions three times per week. Dose exposure will be titrated via increased intensity and duration of exercise as tolerated.

**Self-monitored physical activity and support for sustained behaviour change**

During the 16 week supervised exercise intervention training instructors and intervention staff will support global increase in self-monitored physical activity. Instructors will support the three most effective behaviour change strategies to promote increased physical activity, including participant self-monitoring, goal setting and regular feedback^50,51^. Participants will be provided with accelerometer-based activity monitors, equivalent to the Fitbit or similar device, to allow self-monitoring and target increased physical activity outside of supervised sessions.

The daily physical activity target will be based on the recommended guideline of daily step counts of 10,000 steps per day. Increasing steps counts will be facilitated by titrating a proportional increase from baseline activity to achieve the minimum target of 10,000 steps.

Participants will be consented to allow investigators access to the data collected from wear of the Fitbit or similar device. The information will be used to facilitate feedback on physical activity, goal setting and maintenance of physical activity behaviours.

Following the supervised intervention participants will be encouraged to continue to use their activity monitor and maintain physical activity goals. Remote support will be provided to facilitate feedback and maintenance of physical activity. The intervention team will review continued use of the activity monitor and activity targets at a minimum of 2 weeks. If physical activity is declining or wear time has declined participants with be contacted and offered support to re-engage with behavioural targets. Contact will be maintained via email, text messaging and phone calls. If required, participants will be offered face-to-face support, communication will incorporate motivational strategies to maintain activity goals.

**Comparison Group**

Participants in the control group will be provided with educational materials produced by the British Heart Foundation explaining blood pressure, blood pressure prevention and recommended lifestyle behaviours to maintain heart health.

# SAFETY REPORTING

## Definition of Adverse Event (AE)

Any untoward medical occurrence in a participant to whom exercise intervention has been administered, including occurrences which are not necessarily caused by or related to that intervention.

## Procedures for Recording Adverse Events

All AEs occurring during the trial / or until completion of the final study visit that are observed by the Investigator or reported by the participant, will be recorded on the CRF, whether or not attributed to the exercise intervention.

The following information will be recorded: description, date of onset and end date, severity, assessment of relatedness to exercise intervention, other suspect drug or device and action taken. Follow-up information should be provided as necessary.

The severity of events will be assessed on the following scale: 1 = mild, 2 = moderate, 3 = severe.

AEs considered related to the trial treatment as judged by a medically qualified investigator or the Sponsor will be followed either until resolution, or the event is considered stable.

It will be left to the Investigator’s clinical judgment to decide whether or not an AE is of sufficient severity to require the participant’s removal from treatment. A participant may also voluntarily withdraw from treatment due to what he or she perceives as an intolerable AE. If either of these occurs, the participant must undergo an end of trial assessment and be given appropriate care under medical supervision until symptoms cease, or the condition becomes stable.

## Definition of a Serious Adverse Event

A serious adverse event is any untoward medical occurrence that:

- results in death
- is life-threatening
- requires inpatient hospitalisation or prolongation of existing hospitalisation
- results in persistent or significant disability/incapacity
- consists of a congenital anomaly or birth defect.

Other ‘important medical events’ may also be considered serious if they jeopardise the participant or require an intervention to prevent one of the above consequences.

NOTE: The term "life-threatening" in the definition of "serious" refers to an event in which the participant was at risk of death at the time of the event; it does not refer to an event which hypothetically might have caused death if it were more severe.

## Reporting Procedures for Serious Adverse Events

Safety reporting will be from baseline to the 18 month follow up visit.

Adverse events may also be recorded by the participant using the trial telephone number or email. Participants will be directly asked about adverse events at each study visit.

A serious adverse event (SAE) occurring to a participant should be reported to the REC that gave a favourable opinion of the study where in the opinion of the Chief Investigator the event was ‘related’ (resulted from administration of any of the research procedures) and ‘unexpected’ in relation to those procedures. Reports of related and unexpected SAEs should be submitted within 15 working days of the Chief Investigator becoming aware of the event, using the HRA [report of serious adverse event](http://www.nres.npsa.nhs.uk/docs/forms/Safety_Report_Form_(non-CTIMPs).doc) form (see HRA website).

## Blood Draw and IV Placement

Common risks associated with phlebotomy or cannula insertion are pain during the procedure and bruising (with associated pain afterwards). These risks will be minimised by ensuring that all staff are fully trained in phlebotomy. Bruising after the event will also be reduced by promptly applying pressure on the puncture site after the needle/cannula is withdrawn. All participants will be fully informed about these risks in the Participant Information Leaflet. Though uncommon (<1%) the worry associated with taking blood may cause some participants to feel unwell or faint before, during or after the procedure. The risk associated with this will be reduced by having an adequately equipped facility for performing the procedure (see above) and having a staff member trained in basic life support. Although phlebotomy is a very safe procedure, it does create a puncture wound on the skin which may very rarely lead to infection around the puncture site. The risk of this will be minimised by ensuring strict hygiene during the procedure and by not recruiting participants who are at increased risk of infection. In the event that a participant reports symptoms of an infection (local redness, swelling, pain or discharge of pus) they should be referred to their GP or to A+E urgently.

Risk to Researchers/Other Staff: Taking blood carries a risk of needle stick injury to the phlebotomist, which in turn carries a risk of exposure to blood borne infections. This risk will be minimised by a) ensuring staff are adequately trained, b) ensuring staff have been vaccinated against, and show immunity to Hepatitis B and c) having a local policy for needle stick injury which describes the process of being assessed for and receiving post exposure prophylaxis.

## MRI

MRI is a safe and non-invasive technique with no known risk when appropriately supervised. It does not involve ionising radiation (X-rays). Potential participants with ferromagnetic objects in their bodies or with implanted devices which can be damaged by the MRI magnet will be excluded. All participants entering the scanner room are screened for such objects. Doctors who are Advanced Life Support trained will be available during scans and the site is fully equipped for resuscitation (including defibrillation) in the unlikely event of a medical emergency during scanning.

While most people do not experience discomfort in a MRI environment, the enclosed space of the scanner can potentially feel uncomfortable. Discomfort from lying still for a long period of time will be minimised with comfortable padding and positioning. People with a history of severe claustrophobia would be excluded from participation in the study. Participants will be informed of the possibility of dizziness in the information sheet, and by the operator before they go in the scanner. If the participant does experience dizziness, they may choose to temporarily halt the table movement, they may choose to continue into the centre of the scanner where sensations of dizziness start going away, or they may choose to stop all procedures and come out of the scanner (either temporarily or to withdraw from the study). Participants will be given a chance to see the scanner before the study starts. All participants would be introduced carefully to the scanner and allowed to leave at any stage, should they wish to do so. Once in the scanner, participants would be able to indicate immediately if they wish the scanning to cease by squeezing a bulb placed in their hands, or by verbal request. As the MRI scanner is noisy, participants will be provided with ear plugs and protective headphones to reduce noise and aid communication between them and the investigators. It is important that these are fitted correctly to protect participant’s ears.

## Exercise Training

Exercise training is safe and the established global benefits to health and well-being outweigh negative complications and risks. However, risk of injury, medical complications and serious adverse events are reported in association with exercise participation. Muscle and joint soreness and minor musculoskeletal are common adverse effects of exercise training, however the risk of a serious adverse event including sudden cardiac death or non-fatal cardiac event is reported as below 0.01 per 10000 hours of participation. The following injuries and medical complications may be expected in association with exercise training:

| Accidental Injury | Fall (resulting in admission)  Head injury (resulting in admission)  Road traffic or pedestrian injury |
| --- | --- |
| Allergy | Insect bite  Seasonal allergy exposure |
| Cardiovascular | Exertional Hypotension  Tachycardia  Arrhythmia  Bradycardia  Sudden cardiac event and death |
| Dermatological | Abrasions and Blisters  Dry skin  Fungal infection |
| Gastro-intestinal | Dehydration  Irritable bowel  Increased bowel frequency  Gastro-intestinal blood loss  Gastrosphageal reflux |
| Gynaecology | Irregular menstrual cycle |
| Musculoskeletal | Muscle soreness  Muscle injury  Muscle cramps  Joint injury including sprain  Tendon injury or strain  Ligament tears  Joint inflammation or swelling  Fracture |
| Neurological | Back pain  Nerve entrapment  Headache  Pain |
| Renal | Haematuria  Proteinuria  Exercise associated hyponatraemia |
| Respiratory | Upper respiratory tract infection or viral illness  Exercise induced broncho-spasm |
| Thermal | Heat Cramps  Hyperthermia, Heat exhaustion if exercising in hot/humid environments  Heatstroke  Hypothermia if exercising in cold environments |

Participants shall report adverse events through either hard-copy or secure, computer-based reporting forms. All participants will be briefed to directly seek appropriate clinical care as needed for any adverse events.

## Cardiopulmonary Exercise Testing

Cardiopulmonary exercise testing is very safe and is even used in people with heart failure. However, as can occur with all forms of hard exercise, very occasionally some people have significant changes in their heart rate and rhythm that requires medical attention. Although the risk of this happening is small, the test is carried out in a room equipped with emergency monitoring, emergency medications and resuscitation equipment. Medical personnel will be available throughout the testing. Participants' heart rate and electrocardiography (ECG) are monitored throughout the exercise testing and if there are any concerns the exercise test will be stopped.

# STATISTICS AND ANALYSIS

## Description of Statistical Methods

The analysis will be carried out on the basis of intention-to-treat (ITT). This is, after randomisation, participants will be analysed according to their allocated intervention group irrespective of what they actually receive.

Patient demographic characteristics and other baseline information will be summarised by treatment group. Numbers (with percentages) for binary and categorical variables and mean (standard deviation), or median (interquartile or full range) for continuous variables will be presented. Normality of variables will be assessed by visual assessment of the normality curves and the Shapiro-Wilk test. The analysis of the primary outcome will be assessed using analysis of covariance (ANCOVA) adjusting for baseline values and minimisation factors used in the randomisation process. Results will be presented as adjusted mean difference in change in ambulatory blood pressure between randomised groups at 4 months with 95% confidence intervals (CI) and associated two-sided p value. The analysis of secondary outcomes will also be done using analysis of covariance (ANCOVA) to establish a co-variant model to examine the effect of cardiac structure, vascular function and lifestyle behaviours on exercise capacity. Outcomes measured on more than one occasion will be analysed using a mixed effects model. The data collected from the sub-study will be analysed using paired and independent T-test. If the model assumptions are not met and evidence of departure from Normality is observed, transformations of the data will be employed or non-parametric tests will be carried out. Statistical analysis will be carried out using STATA or SPSS statistical software.

## The Number of Participants

***Primary Outcome 24-hour awake ambulatory blood pressure***

Our systematic review and meta-analysis of randomised control trials identified an average decrease of 5.4mmHg in systolic blood pressure (SBP) post-intervention via supervised exercise in young adults with prehypertension (mean systolic blood pressure at baseline 126 mmHg). The post intervention standard deviation of change was 8.7 mmHg. Our available cross-sectional pilot data suggests that change will be higher in the preterm born participants and participants exposed to maternal hypertension during pregnancy. To calculate the sample size we have been conservative and used an estimated change of 5 mmHg following 4 months of intervention. We have used a SD of 11.3 from the pooled SD for ambulatory systolic blood pressure from our cross-sectional pilot data. To observe a treatment effect on systolic blood pressure of 5mmHg, powered to 80% (p=0.05) requires a total sample size of 164 participants. The SD deviation for the pooled 24 hour awake diastolic blood pressure is 8.3 mmHg. To observe a treatment effect on diastolic blood pressure of 5mmHg, powered to 80% (p=0.05) requires a total sample size of 114 participants. The power calculations for systolic blood pressure are used to determine the final sample size, with adjustment to 200 participants to allow for 18% attrition. To ensure primary objectives for the study are answered the study team may recruit additional participants to replace those who drop out of the study.

***Secondary Outcome Measures***

*Angiogenesis*

*We have previously reported differences in angiogenic factors between preterm and full term participants* (S-Eng preterm 5.64± 1.03 vs control 4.06±0.85 ng/mL). 90% power to observe the same differences will be achieved with at least 10 participants per group. The sample size will be powered to over 80% to observe a 10% change in S-Eng in the preterm and full term participants^9^.

*Microvascular Measures*

*Retinal vasculature*

Hanssen *et al* demonstrated a 6% improvement in arteriolar to venular diameter ratio (AVR) increasing from 0.81 (SD 0.05) to 0.86 (SD 0.1) after 10 weeks aerobic exercise in young healthy males[^43^](#_ENREF_43). To replicate this would require a minimum of 40 participants in the training group and 40 participants in the control group. The sample size of 200 participants is powered to detect a 5% change in retinal imaging parameters.

*Angiogenesis*

*We have previously reported differences in angiogenic factors between preterm and full term participants* (S-Eng preterm 5.64± 1.03 vs control 4.06±0.85 ng/mL). 90% power to observe the same differences will be achieved with at least 10 participants per group. The sample size will be powered to over 80% to observe a 10% change in S-Eng in the preterm and full term participants^9^.

MRI Imaging

MR imaging will be performed on half of the participants in each group. In order to avoid selection bias, all study candidates shall be asked if they would like to take part in MR imaging as a standard component of the consent process. All study participants who volunteer and consent for MR imaging and are not contraindicated to MR scanning, shall be offered a place in their MR imaging sub-group until all places are filled. If there are multiple consented volunteers for the last slot, it shall be offered according to participant consent date (earliest to latest) until the place is filled.

*MRI sub-group Cardiac Measures*

50 participants/group provides 85% power to identify 10% change in LVM index (young adult preterm LVMi 66.5+/-10.9 g/m2) and 80% power to observe a change of 7% in end-diastolic volume (mean EDV 72.2+/-9.3ml) and 12% in longitudinal ventricular strain^14,15^.

*MRI sub-group Cerebrovascular Measures*

White matter integrity

In children and adolescents, maintaining high cardiovascular fitness can be associated with between 5% and 10% difference in regional FA[^42^](#_ENREF_42). In older adults, the difference in regional FA between sedentary groups and those that maintain light to moderate activity is between 1 to 2%. With 100 participants in the MRI study, the intervention will be powered to 80% to report between 4% and 7% change between training and control groups.

*Small vessel tortuosity*

Maintaining higher cardiovascular fitness is associated with over a 15% difference in vessel tortuosity compared to lower fitness in older adults[^32^](#_ENREF_32). The study will be powered to 80% to detect 1% improvement in vessel tortuosity in the exercising participants with 100 participants completing MRI imaging (50 training).

Cerebrovascular Perfusion

Mersov et al in a modelling estimation of the sample size required to detect an arterial spin labelling magnetic resonance imaging perfusion abnormality in voxel-wise group analyses^52^ identified a requirement for a minimum of 22 participants for a 15% difference and 37 participants for a more modest 10% difference.

*Structural volumes*

Grey matter volumes are reduced in preterm born adolescents and young adults compared to full born controls. Mullen *et al* report preterm volumes of 659.3 cm3 (SD 52) compared to 700 cm3 (SD 51)[^42^](#_ENREF_42). This study will be powered to detect 6% improvement in preterm grey matter volumes equivalent to increasing volumes to mean values in the full term population

*Blood validation sub-study*

To validate methodology and assess variability a minimum number of 12 participants are needed as recommended for pilot investigations^53^.

## Analysis of Outcome Measures

Descriptive statistics (mean, standard deviation, standard error, range, etc) will be calculated for each outcome for each group. Differences in the primary and secondary outcomes will be compared between intervention and control groups.

Mean changes in blood pressures and cardiovascular fitness will be compared across the population and correlated with cardiovascular endpoints including cardiac structure and function reported from cardiac MRI and echocardiogram.

Demographic and physiological characteristics of the participants will be added to regression models as covariates to explore the determinants of change in blood pressure comparing preterm and full term born participants.

Statistical analysis will be carried out using a computer software package, applying statistical methods to analyse dependent and independent variables within the study database. The variable will be plotted to categorize distribution and assess if the data is normally distributed which will guide the statistical methods. Methods such as the Student T-test will assess for differences between the groups and linear regression analysis will be used to assess if the study variables can explain differences in primary outcome. It is hoped that this will then allow for clear understanding on how the cardiovascular system adapts following exercise training and how remodelling may differ according to birth history and early life exposures. For the sub-study, the statistical analysis will be carried out using a computer software package and data will be analysed using paired and independent t-test. This will access the variability of circulating blood biomarkers within the same participant across both visits and also between each participant.

A detailed statistical analysis plan will be written with our statistical team and will be completed before receipt of the data.

# DATA MANAGEMENT

## Access to Data

Direct access will be granted to authorised representatives from the Sponsor or host institution for monitoring and/or audit of the study to ensure compliance with regulations.

## Data Recording and Record Keeping

The study will comply with the Data Protection Regulation (GDPR). The University of Oxford, as sponsor will act as data controller for the study. De-coded study data will be entered onto a password protected, electronic cloud based eCRF provided by Castor EDC. Castor EDC is an electronic data capture and management system that permits secure multi-site access. The server is based in the UK and it complies with relevant law to ensure that the data is held securely. The participants will be identified by a unique study specific participant ID in any database and will not be identifiable from this. Electronic documents that contain participants’ personal identifying information such as the code-break document will be stored on a secure server with restricted access, and Informed consent forms that contain participants’ name will be stored securely in locked cupboards and will be only be accessible to study staff and authorised personnel. The name and any other identifying detail will not be included in any trial data electronic file. Trial data electronic file in excel document format will be transferred to a STATA or SPSS spreadsheet for analysis.

During the study up until publication stage hard copy data will be stored securely and after analysis this will be archived in a secure archive location with restricted access.

Electronic data will be held on secure network drives/hard disks/ servers on password protected computers within locked offices. Backup copies of files will be made regularly weekly and stored on a different secure server/external hard drive. These back up locations will be subject to the same security principles as the primary locations. When datasets are complete, the primary copy will remain at the study site where it will be transferred onto optical media, e.g. DVD/external drives and undergoes archiving for hard copy data. Any copies leaving the study site will be completely anonymised/de-identified.

Personal data (such as contact details and information which could identify a participant) (except from those who have consented for future approach) will be destroyed as soon as it is practical to do so and no later than 12 months after the end of the study. The personal identifiers (name of participant) contained in consent forms and the code break document will be stored or accessed for up to 7 years after study end, after which time the custodian will agree a date for destruction and it will be destroyed confidentially.

As part of our commitment to maximise patient/service user involvement in research, participants will give consent (optional) for their contact details to be retained. This will be kept securely and independently of the study records. This will allow investigators to contact participants about future ethically approved research.

# QUALITY ASSURANCE PROCEDURES

The trial will be conducted in accordance with the current approved protocol, GCP, relevant regulations and standard operating procedures.

Regular monitoring will be performed according to GCP. Data will be evaluated for compliance with the protocol and accuracy in relation to source documents. Following written standard operating procedures, the monitors will verify that the clinical trial is conducted and data are generated, documented and reported in compliance with the protocol, GCP and the applicable regulatory requirements.

A Trial Steering Committee has been formed to oversee the conduct of the trial. The TSC will consist of the Prof Paul Leeson (Chief Investigator), Prof Charlie Foster, Prof Helen Dawes, and Dr Julia Newton, a study statistician, independent statistician, a Professor of Exercise and/or Cardiovascular Prevention from an independent institution and lay committee member.

The TSC will next meet when trial recruitment commences, within 6 months of recruitment opening and after approximately a third of participants have completed their second visit.

A data monitoring committee is being formed. Rates of recruitment, trial compliance, measurement of primary outcome and study attrition will be review annually by the independent members of the Data Monitoring Committee.

# ETHICAL AND REGULATORY CONSIDERATIONS

## MRI Imaging

MIR is considered safe and uses no ionising radiation. Contraindications to MRI imaging are explained in section 10.6 and in the Participant Information Leaflet and will be made clear to participants during the Informed Consent process and prior to entering the MRI scanning facilities. Any concerns regards the participants’ MRI safety will result in the MRI imaging not being performed.

## Storage of Blood Samples and Images

It will be made clear to participants as part of the informed consent process how images and blood samples they provide will be used for this study and stored.

## Genetic Testing

It is possible that some samples will be used for genetic research. This research may be conducted by the study research team or collaborating research teams. The genetic tests would involve looking at common variations in genes that affect how blood vessels work. We do not propose to test for inherited genetic diseases. There is no evidence to suggest that the results of these genetic studies would have significant implications for the participant.

It will be explained to participants during the consent process that blood samples may be used for genetic testing. It will be explained that the genetic tests would involve looking at common variations in genes that affect how blood vessels work and that tests for inherited genetic diseases will not be performed. Genetic information is kept strictly confidential and results of genetic analysis will not be disclosed to participants or their treating physicians.

## Cardiopulmonary Exercise Testing

The study procedures including the cardiopulmonary exercise testing are explained in the Participant Information Leaflet and will be made clear to the participants as part of the informed consent process. If there are any concerns regarding a participant’s safety during the exercise testing, they will be asked to stop and safety to continue in the trial will be reviewed by trained and qualified personnel. Doctors who are Advanced Life Support trained will be available during CPET testing and the site is fully equipped for resuscitation (including defibrillation) in the unlikely event of a medical emergency during the test.

## Exercise Associated Musculoskeletal Injury

Study participants entering the exercise training arm will be instructed on injury prevention practice including warm-up and stretching exercises. Participants will be encouraged to maintain regular preventive lower limb exercise demonstrated during the supervised sessions. The study team includes training sports and exercise medicine physicians experienced in injury prevention and rehabilitation. If there are any concerns regarding a participant’s safety to continue in the trial secondary to injury, they will be asked to stop training. Safety to continue in the trial will be reviewed by trained and qualified personnel identified in the delegation log and the outcome will be discussed with their primary care team as appropriate.

## Use of Monitoring Devices

The use of blood pressure and physical activity monitoring and the requirement as part of the study procedures to wear and return the devices is clearly explained in the Participant Information Leaflet and will be explained in the informed consent process. Wearing the ABP monitor has no direct risks and causes no pain. They are used routinely in the hospital outpatient setting. The monitor is small and light can be kept in a pocket or worn in waist pouch. Participants are unable to bathe, shower or swim while wearing the monitor as it must not get wet. Also the research team will instruct all participants on how to turn the monitor of whilst driving. These guidelines are also provided to participants on an information sheet when they are fitted with the monitor.

## Incidental Findings

The study is wholly research-oriented, and the images of the heart, liver and brain are for specific research purposes only, and are not suitable for diagnostic opinions. However, although the images are not diagnostic scans, in the event of an abnormality of clear clinical relevance being noted incidentally, a designated clinical specialist would discuss the implications with the participant and with the consent of the participant, arrange for further investigations as necessary. The participant’s general practitioner would also be informed of the abnormality with the consent of the participant, so that on-going medical care can be arranged.

## Declaration of Helsinki

The Investigator will ensure that this study is conducted in accordance with the principles of the Declaration of Helsinki.

## Guidelines for Good Clinical Practice

The Investigator will ensure that this study is conducted in accordance with relevant regulations and Good Clinical Practice.

## Approvals

The protocol, informed consent form, participant information sheet and any proposed advertising material will be submitted to an appropriate HRA Research Ethics Committee (REC), and host institution(s) for written approval. The Investigator will submit and, where necessary, obtain approval from the above parties for all substantial amendments to the original approved documents.

## Reporting

The CI shall submit once a year throughout the study or on request, an Annual Progress report to the REC Committee, host organisation and Sponsor. In addition, an End of Study notification and final report will be submitted to the same parties.

## Participant Confidentiality

The study staff will ensure that the participants’ anonymity is maintained. The participants will be identified only by initials and a participants ID number on the CRF/eCRF and in the electronic database. All documents will be stored securely and only accessible by study staff and authorised personnel. The study will comply with the General Data Protection Regulation (GDPR) and associated Data Protection Act 2018, which requires data to be anonymised as soon as it is practical to do so.

## Expenses and Benefits

Reasonable travel expenses for attending the study visits will be reimbursed on production of receipts, or a mileage allowance provided as appropriate. If participants require food or refreshments following the study they will be offered reasonable reimbursement for products purchased in the hospital facilities on production of receipts. As a thank you for their participation all volunteers will be provided with a £50 monetary gift or gift voucher for completing baseline measures and follow-up visits at 16 weeks and 52 weeks. In addition, participants in the control group will receive an approximate monetary equivalent of 3 months gym membership (£100) at the end of the TEPHRA trial. No reimbursement will be provided for participants for TEPHRA blood validation study.

## Other Ethical Considerations

Participants who do not have the capacity to provide informed consent or who lose the capacity after entering the study will either not be recruited or will be withdrawn.

# FINANCE AND INSURANCE

## Funding

The study is funded by the Wellcome Trust, grant number 105741/Z/14/Z and BHF Project Grant no. PG/17/13/32860.

## Insurance

The University has a specialised insurance policy in place which would operate in the event of any participant suffering harm as a result of their involvement in the research (Newline Underwriting Management Ltd, at Lloyd’s of London)

# PUBLICATION POLICY

The drafting and reviewing of the manuscripts, abstracts, press releases and all other publications arising from the study will be exclusively the responsibility of the study investigators. Authors will acknowledge that the study was funded by the Wellcome Trust. Authorship will be determined in accordance with the ICMJE guidelines and other contributors will be acknowledged

# REFERENCES

1 World Health Organisation. World Health Statistics 2012. 2012.

2 Scarborough P, Bhatnagar P, Wickramasinghe K, Smolina K, Mitchell C. Coronary heart disease statistics 2010 edition. *Br Hear Found* 2010; : 21.

3 Nguyen QC, Tabor JW, Entzel PP, *et al.* Discordance in national estimates of hypertension among young adults. *Epidemiology* 2011; **22**: 532–41.

4 Lewington S, Clarke R, Qizilbash N, Peto R, Collins R. Age-specific relevance of usual blood pressure to vascular mortality: a meta-analysis of individual data for one million adults in 61 prospective studies. *Lancet* 2002; **360**: 1903–13.

5 Kajantie E, Hovi P. Is very preterm birth a risk factor for adult cardiometabolic disease? *Semin Fetal Neonatal Med* 2014; **19**: 112–7.

6 de Jong F, Monuteaux MC, van Elburg RM, Gillman MW, Belfort MB. Systematic review and meta-analysis of preterm birth and later systolic blood pressure. *Hypertension* 2012; **59**: 226–34.

7 Crump C, Winkleby M a, Sundquist K, Sundquist J. Risk of hypertension among young adults who were born preterm: a Swedish national study of 636,000 births. *Am J Epidemiol* 2011; **173**: 797–803.

8 Davis E, Lazdam M, Lewandowski A. Cardiovascular risk factors in children and young adults born to preeclamptic pregnancies: a systematic review. *Pediatrics* 2012; **129**: 1552–61.

9 Lewandowski AJ, EF D, G Y, *et al.* Elevated blood pressure in preterm-born offspring associates with a distinct antiangiogenic state and microvascular abnormalities in adult life. *Hypertension* 2015; **65**: 607–14.

10 Bonamy A-KE, Källén K, Norman M. High blood pressure in 2.5-year-old children born extremely preterm. *Pediatrics* 2012; **129**: e1199–204.

11 Parkinson J, MJ H, C G, S S, N M. Preterm birth and the metabolic syndrome in adult life: a systematic review and meta-analysis. *Pediatrics* 2013; **131**: e1240.

12 Davis EF, Lewandowski AJ, Aye C, *et al.* Clinical cardiovascular risk during young adulthood in offspring of hypertensive pregnancies: insights from a 20-year prospective follow-up birth cohort. *BMJ Open* 2015; **5**: e008136.

13 Siebel AL, Carey AL, Kingwell B a. Can exercise training rescue the adverse cardiometabolic effects of low birth weight and prematurity? *Clin Exp Pharmacol Physiol* 2012; **39**: 944–57.

14 Lewandowski AJ, Augustine D, Lamata P, *et al.* Preterm heart in adult life: cardiovascular magnetic resonance reveals distinct differences in left ventricular mass, geometry, and function. *Circulation* 2013; **127**: 197–206.

15 Lewandowski AJ, Bradlow WM, Augustine D, *et al.* Right ventricular systolic dysfunction in young adults born preterm. *Circulation* 2013; **128**: 713–20.

16 Spence AL, Naylor LH, Carter HH, *et al.* A prospective randomised longitudinal MRI study of left ventricular adaptation to endurance and resistance exercise training in humans Name and address for corresponding author. *J Physiol* 2011; **15**: 5443–52.

17 Andersen LJ, Randers MB, Hansen PR, *et al.* Structural and functional cardiac adaptations to 6 months of football training in untrained hypertensive men. *Scand J Med Sci Sports* 2014; : 1–9.

18 Murias JM, Kowalchuk JM, Ritchie D, Hepple RT, Doherty TJ, Paterson DH. Adaptations in capillarization and citrate synthase activity in response to endurance training in older and young men. *J Gerontol A Biol Sci Med Sci* 2011; **66**: 957–64.

19 Hoier B, Nordsborg N, Andersen S, *et al.* Pro- and anti-angiogenic factors in human skeletal muscle in response to acute exercise and training. *J Physiol* 2012; **590**: 595–606.

20 Gielen S, Schuler G, Adams V. Cardiovascular effects of exercise training: molecular mechanisms. *Circulation* 2010; **122**: 1221–38.

21 Vijayaraghavan K, Deedwania P. Renin-Angiotensin-Aldosterone Blockade for Cardiovascular Disease Prevention. *Cardiol Clin* 2011; **29**: 137–56.

22 Lin X, Zhang X, Guo J, *et al.* Effects of Exercise Training on Cardiorespiratory Fitness and Biomarkers of Cardiometabolic Health: A Systematic Review and Meta-Analysis of Randomized Controlled Trials. *J Am Heart Assoc* 2015; **4**: 1–29.

23 Hespel P, Lijnen P, Van Hoof R, *et al.* Effects of physical endurance training on the plasma renin-angiotensin-aldosterone system in normal man. *J Endocrinol* 1988; **116**: 443–9.

24 Karumanchi SA, Maynard SE, Stillman IE, Epstein FH, Sukhatme VP. Preeclampsia: A renal perspective. *Kidney Int* 2005; **67**: 2101–13.

25 Bertagnolli M, Casali KR, De Sousa FB, *et al.* An orally active angiotensin-(1-7) inclusion compound and exercise training produce similar cardiovascular effects in spontaneously hypertensive rats. *Peptides* 2014; **51**: 65–73.

26 Chen Y-P, Lu Y-P, Li J, *et al.* Fetal and maternal angiotensin (1-7) are associated with preterm birth. *J Hypertens* 2014; **32**: 1833–41.

27 Emdin M, Fatini C, Mirizzi G, *et al.* Biomarkers of activation of renin-angiotensin-aldosterone system in heart failure: how useful, how feasible? *Clin Chim Acta* 2015; **443**: 85–93.

28 Kajantie E, Eriksson JG, Osmond C, Thornburg K, Barker DJP. Pre-eclampsia is associated with increased risk of stroke in the adult offspring: the Helsinki birth cohort study. *Stroke* 2009; **40**: 1176–80.

29 Malamateniou C, Counsell SJ, Allsop JM, *et al.* The effect of preterm birth on neonatal cerebral vasculature studied with magnetic resonance angiography at 3 Tesla. *Neuroimage* 2006; **32**: 1050–9.

30 Ballabh P, Braun A, Nedergaard M. Anatomic analysis of blood vessels in germinal matrix, cerebral cortex, and white matter in developing infants. *Pediatr Res* 2004; **56**: 117–24.

31 Khwaja O, Volpe JJ. Pathogenesis of cerebral white matter injury of prematurity. *Arch Dis Child - Fetal Neonatal Ed*  2008; **93** : F153–61.

32 Patton N, Aslam T, MacGillivray J, Pattie a, Deary I, Dhillon B. Retinal vascular image analysis as a potential screening tool for cerebrovascular disease. *J Anat* 2005; **206**: 318–48.

33 Poels MMF, Steyerberg EW, Wieberdink RG, *et al.* Assessment of cerebral small vessel disease predicts individual stroke risk. *J Neurol Neurosurg Psychiatry*  2012; **83** : 1174–9.

34 Hilal S, Ong Y-T, Cheung CY, *et al.* Microvascular network alterations in retina of subjects with cerebral small vessel disease. *Neurosci Lett* 2014; **577**: 95–100.

35 Schmidt W, Endres M, Dimeo F, Jungehulsing GJ. Train the vessel, gain the brain: Physical activity and vessel function and the impact on stroke prevention and outcome in cerebrovascular disease. *Cerebrovasc Dis* 2013; **35**: 303–12.

36 Wen CP, Wai JPM, Tsai MK, *et al.* Minimum amount of physical activity for reduced mortality and extended life expectancy: a prospective cohort study. *Lancet* 2011; **378**: 1244–53.

37 Ang E., Wong PT., Moochhala S, Ng Y. Neuroprotection associated with running: is it a result of increased endogenous neurotrophic factors? *Neuroscience* 2003; **118**: 335–45.

38 Ding Y, Li J, Luan X, *et al.* Exercise pre-conditioning reduces brain damage in ischemic rats that may be associated with regional angiogenesis and cellular overexpression of neurotrophin. *Neuroscience* 2004; **124**: 583–91.

39 Bullitt E, Rahman FN, Smith JK, *et al.* The effect of exercise on the cerebral vasculature of healthy aged subjects as visualized by MR angiography. *Am J Neuroradiol* 2009; **30**: 1857–63.

40 Black JE, Isaacs KR, Anderson BJ, Alcantara AA, Greenough WT. Learning causes synaptogenesis, whereas motor activity causes angiogenesis, in cerebellar cortex of adult rats. *Proc Natl Acad Sci*  1990; **87** : 5568–72.

41 Voss MW, Vivar C, Kramer AF, van Praag H. Bridging animal and human models of exercise-induced brain plasticity. *Trends Cogn Sci* 2013; **17**: 525–44.

42 Hanssen H, Nickel T, Drexel V, *et al.* Exercise-induced alterations of retinal vessel diameters and cardiovascular risk reduction in obesity. *Atherosclerosis* 2011; **216**: 433–9.

43 Bhatia LS, Curzen NP, Calder PC, Byrne CD. Non-alcoholic fatty liver disease: a new and important cardiovascular risk factor? *Eur Heart J* 2012; **33**: 1190–200.

44 Kantartzis K, Thamer C, Peter A, *et al.* High cardiorespiratory fitness is an independent predictor of the reduction in liver fat during a lifestyle intervention in non-alcoholic fatty liver disease. *Gut* 2009; **58**: 1281–8.

45 Keating SE, Hackett DA, George J, Johnson NA. Exercise and non-alcoholic fatty liver disease: A systematic review and meta-analysis. *J Hepatol* 2012; **57**: 157–66.

46 Johnson NA, George J. Fitness versus fatness: moving beyond weight loss in nonalcoholic fatty liver disease. *Hepatology* 2010; **52**: 370–81.

47 NICE. Hypertension: Clinical management of primary hypertension in adults (NICE clinical guideline 127). *NICE* 2011; : 38.

48 Cornelissen V a, Smart N a. Exercise training for blood pressure: a systematic review and meta-analysis. *J Am Heart Assoc* 2013; **2**: e004473.

49 Cornelissen V a, Buys R, Smart N a. Endurance exercise beneficially affects ambulatory blood pressure: a systematic review and meta-analysis. *J Hypertens* 2013; **31**: 639–48.

50 Michie S, Abraham, Charles; Whittington C, McAteer J, Gupta S. Effective techniques in healthy eating and physical activity interventions: A meta-regression. *Heal Psychol* 2009; **28**: 690–701.

51 Greaves CJ, Sheppard KE, Abraham C, *et al.* Systematic review of reviews of intervention components associated with increased effectiveness in dietary and physical activity interventions. *BMC Public Health* 2011; **11**: 119.

52 A.M. M, D.E. C, M.A. C, S.E. B, B.J. M. Estimating the sample size required to detect an arterial spin labelling magnetic resonance imaging perfusion abnormality in voxel-wise group analyses. *J Neurosci Methods* 2015; **245**: 169–77.

53 Julious SA. 185Sample size of 12 per group rule ofthumb for a pilot study. *Pharmaceut Statist* 2005; **4**: 287–291.

# APPENDIX A: STUDY FLOW CHART


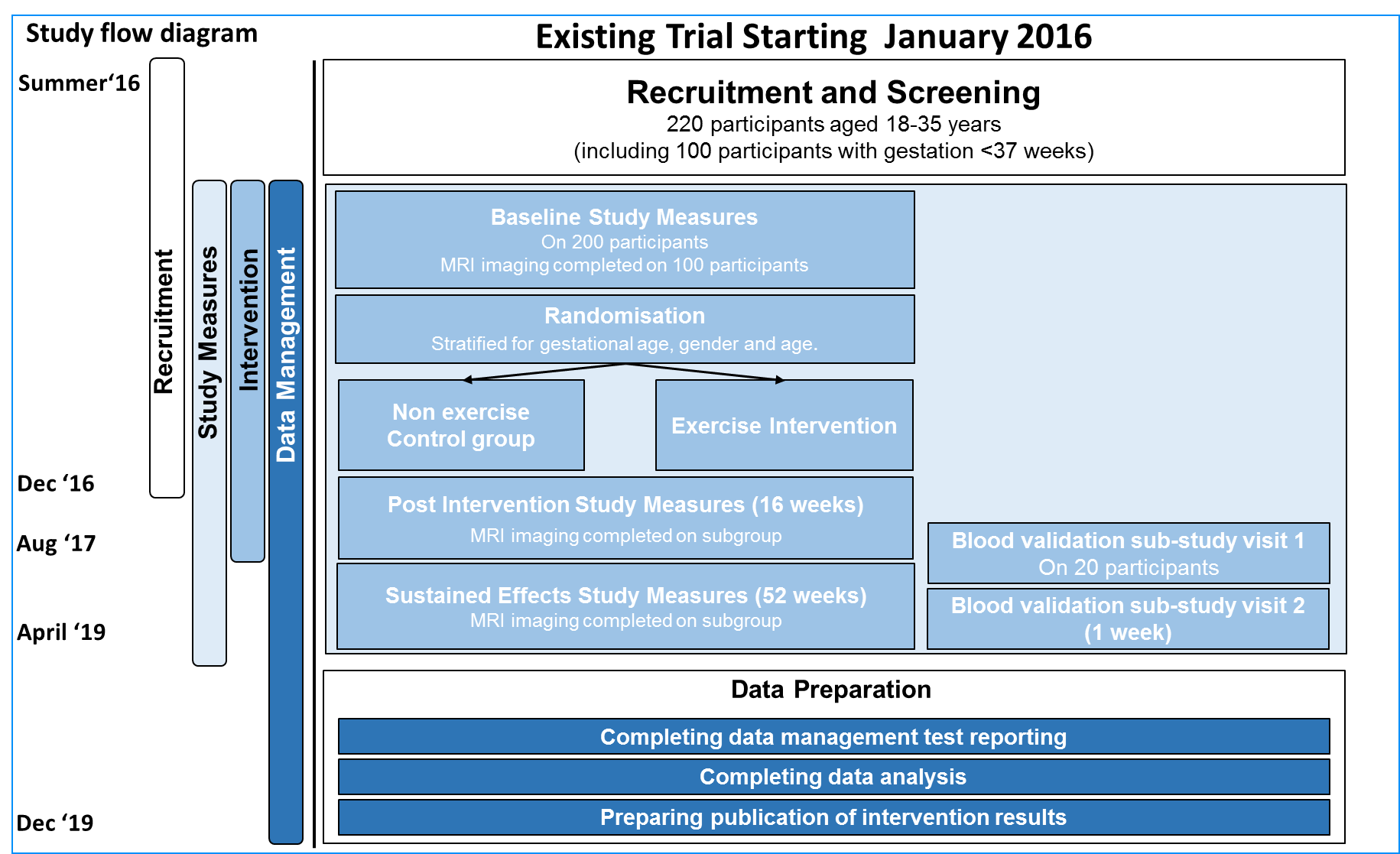


# APPENDIX B: STUDY STAGES and STUDY MEASURES

**TEPHRA Study:**

**
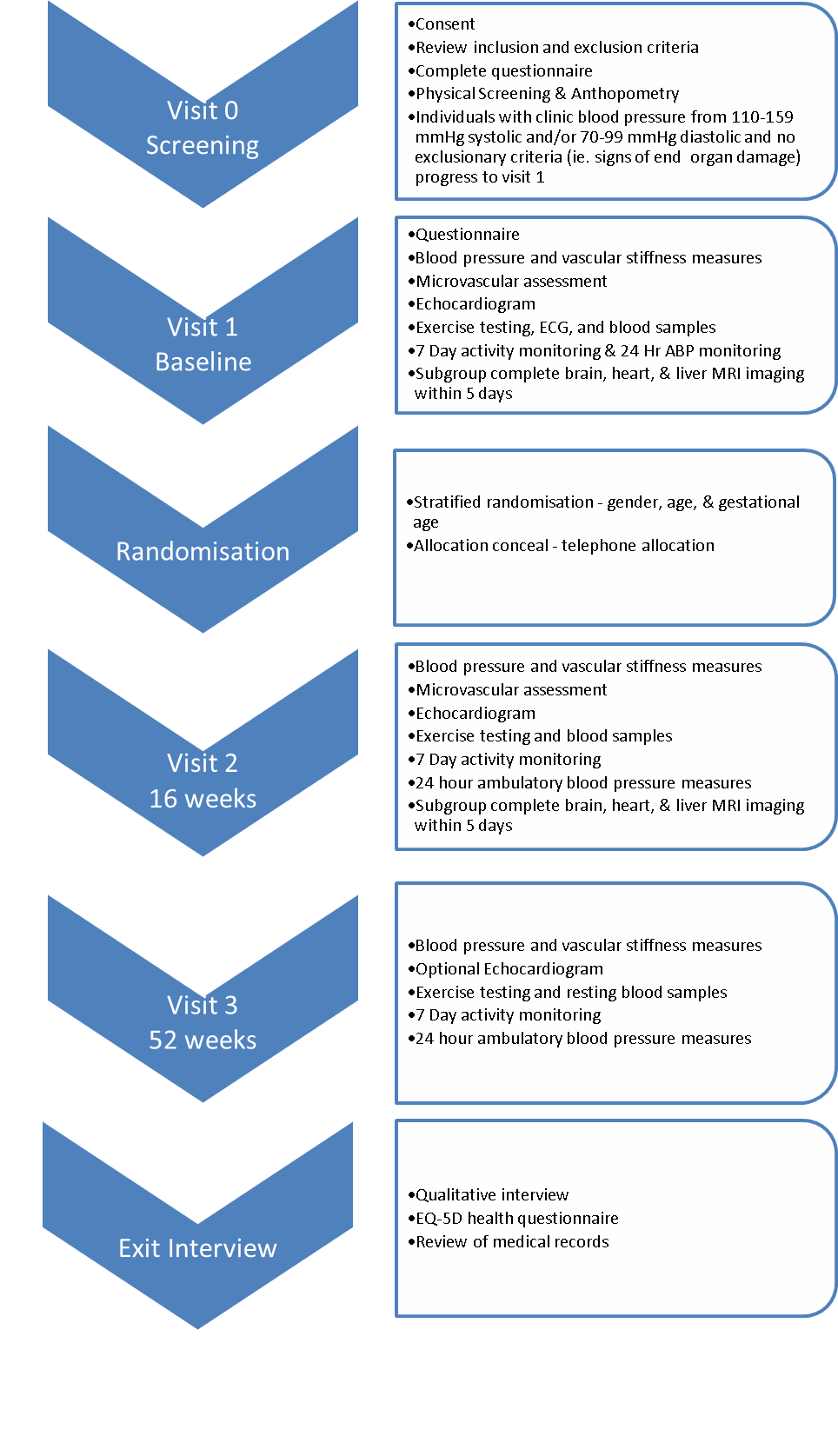
**

**TEPHRA Blood validation sub-study:**

**
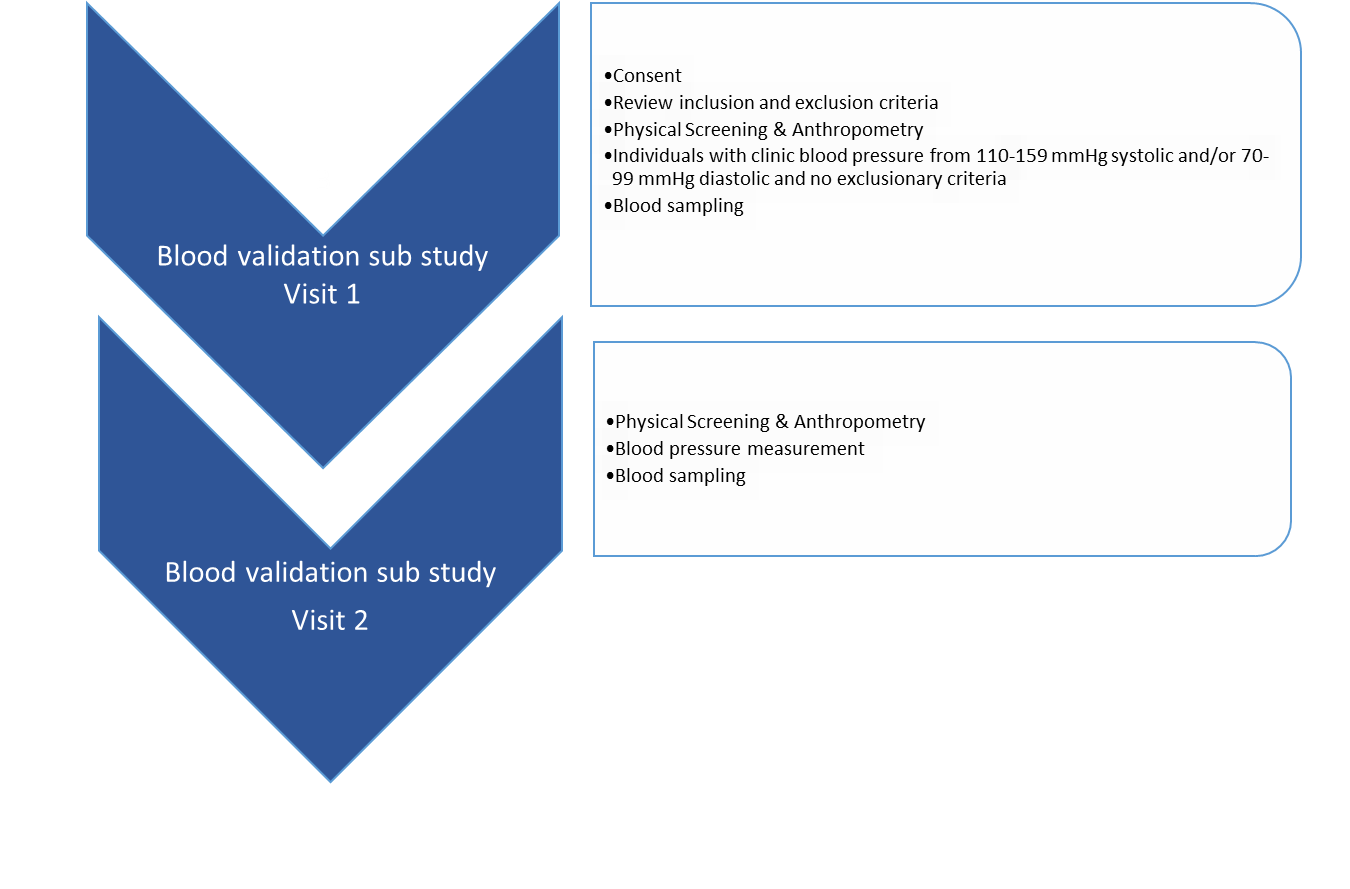
**

# APPENDIX C: SCHEDULE OF MEASURES and TIME ALLOCATION

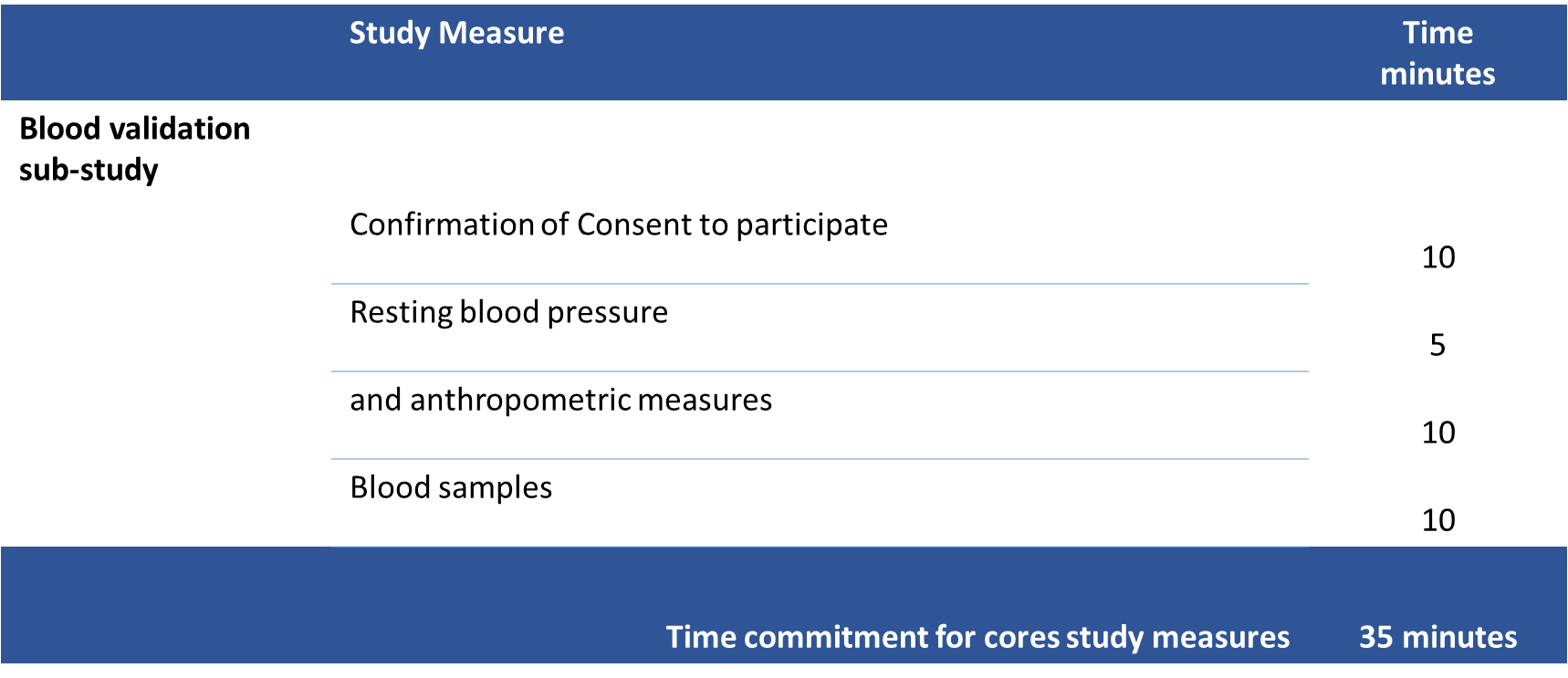


# APPENDIX D: GANTT CHART


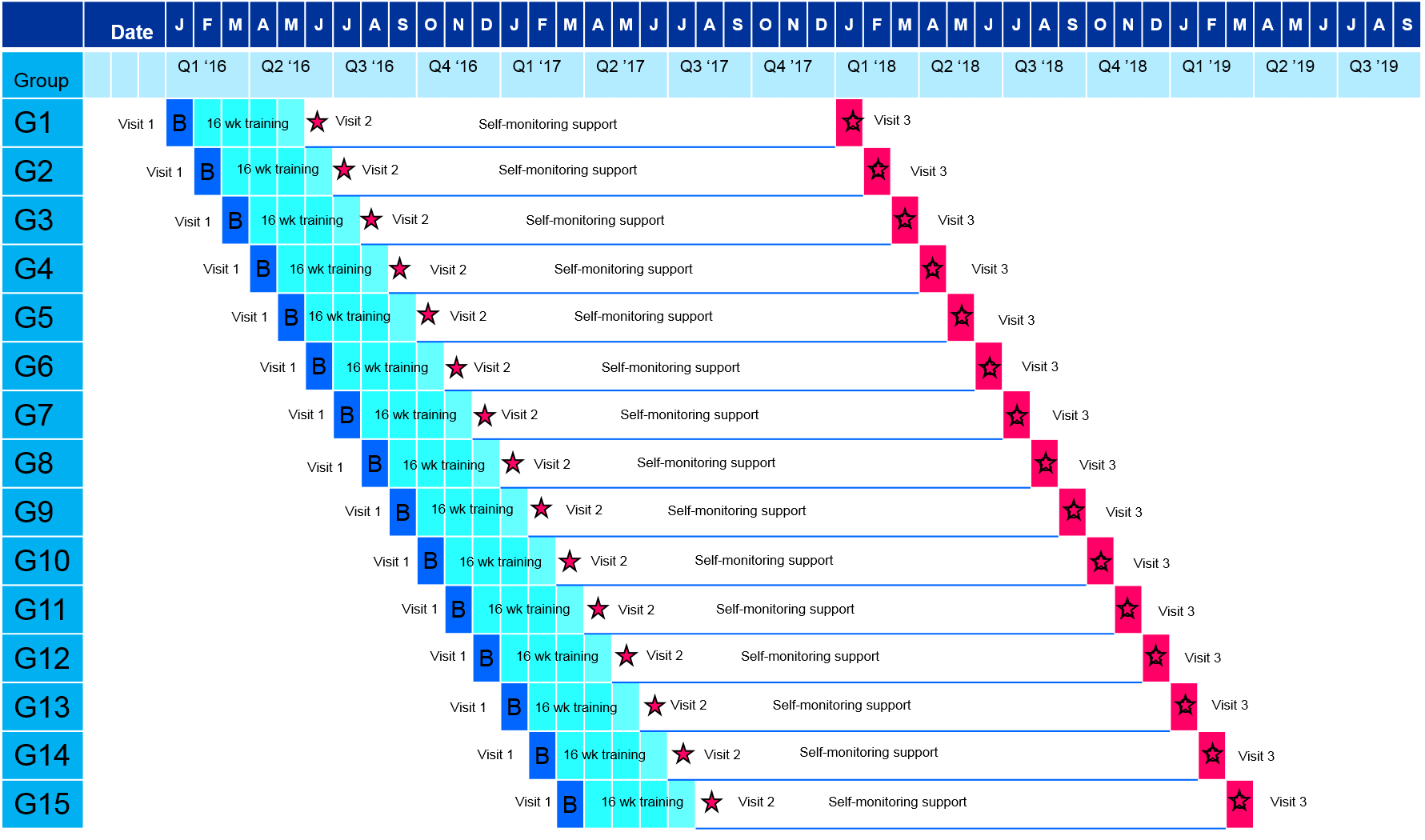


# APPENDIX E: PHYSICAL ACTIVITY INTERVENTION

# APPENDIX F: AMENDMENT HISTORY

| **Amendment No.** | **Protocol Version No.** | **Date issued** | **Author(s) of changes** | **Details of Changes made** |
| --- | --- | --- | --- | --- |
| 1 | 1.0 | 11 April 2016 |  | Invitation Letter to GP, not submitted with original application when applying for REC approval |
| 2 | 2.0 | 25 Apr 2017 | O. Huckstep  &  W. Williamson | Protocol amended to   - Move initial 24 hour blood pressure monitoring and ECG from screening to baseline visit. - Reduce re-occurrence of selected secondary outcome measures (e.g., microvascular assessment, gait analysis and post exercise blood samples from the core study measures and MRI imaging from the subgroup) at the 52 week follow-up visit. - Refine statistical analysis. - Study member names updated. |
| 3 | 2.0 | 09 May 2018 |  | Additional text for advertisements (means of advertising already approved, only additional text to include). |
| 4 | 3.0 | 09 April 2019 | C. Tan | Protocol amended to   - Include a sub-population of 20 healthy individual for blood validation sub study. - Add a further objective in relation to the sub study. - The transfer of blood samples from YACTH and EXPRESSO study. - Refine data management. - Study member name updated. - Funder added |
| 5 | 3.1 | December 2020 | R Jones | Extension of end date to 31^st^ May 2021 |
